# Ubiquitin-dependent proteasomal degradation of small hepatitis B virus surface antigen mediated by TRIM21 and antagonized by OTUD4

**DOI:** 10.1128/jvi.02309-24

**Published:** 2025-04-25

**Authors:** Shuxiang Wu, Zhihan Chen, Zhengqian Zhang, Jing Xu, Hang Li, Mengxian Lin, Wenjie Xie, Yan Chen, Xinjian Lin, Xu Lin

**Affiliations:** 1 Key Laboratory of Gastrointestinal Cancer (Fujian Medical University), Ministry of Education, Fuzhou, China; 2 Fujian Key Laboratory of Tumor Microbiology, Department of Medical Microbiology, Fujian Medical University 74551, Fuzhou, China; Wake Forest University School of Medicine, Winston-Salem, North Carolina, USA

**Keywords:** small hepatitis B virus surface antigen, ubiquitination, TRIM21, OTUD4, hepatitis B virus

## Abstract

The small hepatitis B surface antigen (SHBs) is the most abundant hepatitis B virus (HBV) protein in individuals infected with HBV, and clearance of HBV surface antigen, which is primarily composed of SHBs, is considered a surrogate biomarker for achieving a functional cure of chronic HBV. Understanding SHBs degradation is crucial for its elimination and targeted eradication strategies. This study demonstrates that SHBs undergoes degradation via a ubiquitin/proteasome pathway, primarily through K48-linked ubiquitination, with K122 as the critical ubiquitination site. Utilizing immunoprecipitation and mass spectrometry, we identified TRIM21 (an E3 ubiquitin ligase) and OTUD4 (a deubiquitinase) as key regulators of SHBs. We verified the direct interaction between SHBs and TRIM21’s coiled-coil domain, as well as the N-terminal amino acids 1–180 of OTUD4, using coimmunoprecipitation and glutathione S-transferase (GST) pull-down assays in both *in vivo* and *in vitro* settings. TRIM21 was observed to reduce SHBs stability and abundance by promoting its polyubiquitination, whereas OTUD4 acted to negate the effects of TRIM21-induced polyubiquitination, thereby stabilizing and increasing the levels of SHBs. Notably, TRIM21-mediated degradation of SHBs substantially impaired subviral particle and virion production and its biological activities such as migratory and angiogenic capabilities, opposite to the effect produced by the introduction of OTUD4. These findings suggest that TRIM21 and OTUD4 modulate SHBs protein stability and function through a ubiquitination-dependent proteasomal pathway, offering new insights into clearing SHBs and intervening in the progression of HBV-related liver diseases.

**IMPORTANCE:**

The small hepatitis B surface antigen (SHBs) is a key structural component of the hepatitis B virus (HBV) virion and subviral particles and is the most abundant HBV protein in individuals with chronic infection. Gaining a better understanding of its degradation pathways may help inform strategies to reduce SHBs levels and potentially support the design of targeted therapies. However, the specific mechanisms and processes involved in the degradation of SHBs remain largely unexplored. This study reveals that SHBs is degraded via the ubiquitin/proteasome pathway, specifically through K48-linked ubiquitination at the K122 site. TRIM21 promotes SHBs degradation by enhancing its polyubiquitination, while OTUD4 stabilizes SHBs by counteracting TRIM21’s effects. TRIM21 reduces SHBs stability, subviral particle and virion production, and its related biological activities, whereas OTUD4 stabilizes SHBs, promoting its accumulation. These findings highlight the roles of TRIM21 and OTUD4 in regulating SHBs stability and function, offering new insights into potential interventions for HBV-related liver diseases.

## INTRODUCTION

Hepatitis B virus (HBV) infection is a major global health issue, leading to cirrhosis and liver cancer. It affects about 296 million people worldwide, causing around 1 million deaths each year (1). HBV contains a 3.2 kb DNA that encodes several proteins, including HBV e antigen (HBeAg), HBV core antigen, HBV DNA Polymerase (DNA Pol), HBV X proteins (HBx), and HBV surface antigen (HBsAg), which has large, middle, and small components (LHBs, MHBs, and SHBs) (2). Chronic HBV infection significantly increases the risk of developing hepatocellular carcinoma (HCC) by up to 100 times (3). The challenge in curing HBV lies in the presence of covalently closed circular DNA and integrated HBV DNA within the host cells. However, clearing the HBsAg, especially SHBs, suppresses HBV replication and reduces the risk of developing HCC, making it a “functional cure” for HBV (4, 5).

SHBs, the most abundant HBV viral protein, plays a major role in the virus’s replication and immune system evasion (6–8). It activates Golgi protein 73, which suppresses a specific immune factor, aiding HBV replication (6). SHBs also causes stress in the endoplasmic reticulum (ER), triggering various cellular processes including autophagy (9), angiogenesis (10), and metastasis (11). Moreover, SHBs promotes hepatic gluconeogenesis by activating the cyclic AMP (cAMP)/protein kinase A (PKA)/cAMP-responsive element binding protein (CREB) signaling pathway and subsequently inducing the expression of gluconeogenic genes (12). It also interacts with other proteins such as cyclophilin to induce inflammation, contributing to HBV infection (13). Given its multiple critical functions in HBV life cycle and disease-causing capability, the suppression of SHBs by antibodies can hinder HBV-induced hepatocarcinogenesis (14). Focusing on breaking down SHBs could be an effective strategy for achieving a “functional cure” in HBV treatment. Therefore, a deep understanding of the SHBs degradation pathways and mechanisms will aid in developing therapeutic methods to clear SHBs, which is of significant importance for more effectively treating HBV-related liver diseases.

The degradation of intracellular proteins predominantly occurs via pathways that can be either ubiquitin-dependent or independent, utilizing autophagy-lysosome systems or proteasomes. In this context, the ubiquitination process of proteins is governed by reversible modifications enacted by E3 ubiquitin ligases and deubiquitinating enzymes (15). Studies have shown that several HBV viral proteins are regulated by E3 ubiquitin ligases or deubiquitinating enzymes, including the Pol protein binding with E3 ubiquitin ligase TRIM21 or with c-Abl for degradation via the ubiquitin-proteasome pathway, thus inhibiting HBV replication (16, 17). HBc protein interacts with E3 ubiquitin ligase NIRF to mediate its degradation, thereby suppressing HBV replication (18). HBx can be degraded by the polyubiquitin-dependent proteasome pathway mediated by E3 ubiquitin ligase Siah-1, and it can also inhibit degradation through a ubiquitin-independent manner by directly binding to the proteasome through interaction with the deubiquitinating enzyme VCPIP1 (19). Yet, the degradation pathway of SHBs and whether it is ubiquitin-dependent or not remains to be determined.

In this study, we demonstrate that E3 ubiquitin ligase TRIM21 and deubiquitinating OTUD4 directly interact with the SHBs protein, marking it for breakdown through the ubiquitination process and subsequent proteasomal degradation. Increasing the levels of TRIM21 and reducing OTUD4 activity could enhance the breakdown of SHBs protein. This approach holds potential as a promising strategy for achieving a “functional cure” in the treatment of HBV in clinical settings.

## RESULTS

### SHBs undergoes a process of ubiquitination and proteasome degradation

**Fig 1:**
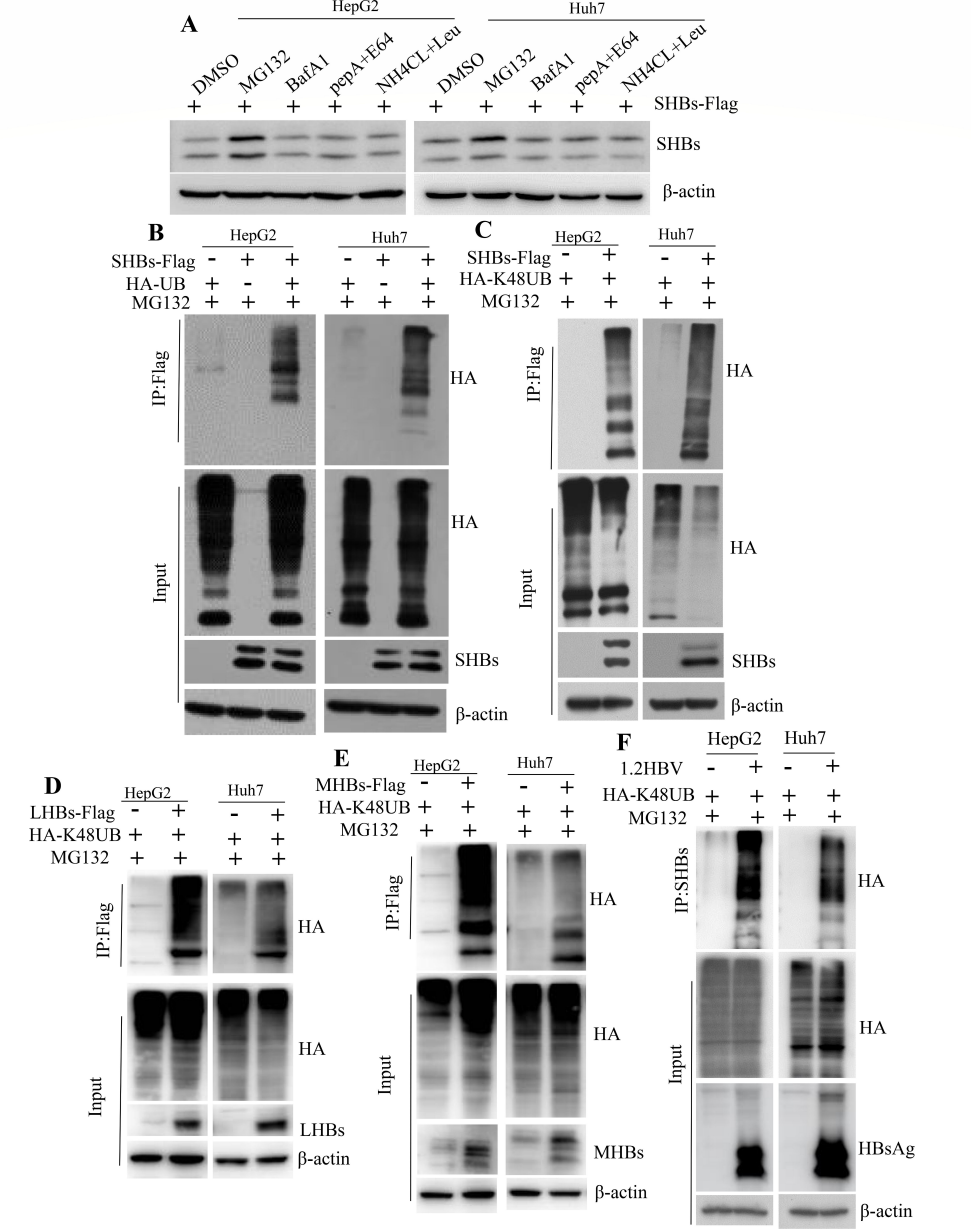
SHBs undergoes ubiquitination and is degraded in a proteasome-dependent manner. (A) Cells transfected with plasmid encoding SHBs-Flag were treated with the proteasome inhibitor MG132 (20 µM), lysosomal protease inhibitors E64D + pepstatin (10 mM), leupeptin + NH4Cl (100 nM), or the autophagy inhibitor bafilomycin A1 (100 nM) for 8 hours, and SHBs expression levels were assessed using immunoblotting with SHBs-specific antibodies. (B, C) Cells co-transfected with plasmids encoding SHBs-Flag and either HA-UB or HA-K48UB were treated with MG132 (20 µM) for 8 hours and then underwent the assessment of SHBs ubiquitination (**B**) and K48-linked polyubiquitination (**C**) via immunoprecipitation using anti-FLAG M2 affinity gel and subsequent immunoblot analysis. (D, E) Cells co-transfected with plasmids encoding HA-K48UB and either LHBs-Flag or MHBs-Flag were treated with MG132 (20 µM) for 8 hours. Following treatment, the K48-linked polyubiquitination of LHBs (**D**) and MHBs (**E**) was assessed through immunoprecipitation using anti-FLAG M2 affinity gel, followed by immunoblot analysis. (F) Cells co-transfected with plasmids encoding HA-K48UB and 1.2HBV DNA were similarly treated with MG132 (20 µM) for 8 hours. The K48-linked polyubiquitination of HBsAg was then evaluated via immunoprecipitation using anti-SHBs affinity gel, followed by immunoblot analysis.

The processes of SHBs degradation and its underlying mechanisms remain largely uncharted. We aimed to determine whether SHBs protein gets degraded by the proteasome or through autophagy-lysosome pathways. We treated SHBs-expressing HCC cells with various inhibitors including proteasome inhibitor MG132, lysosomal protease inhibitors (E64D + pepstatin and leupeptin + NH4Cl), or an autophagy inhibitor (bafilomycin A1). The results showed that the degradation of SHBs was effectively hindered by the proteasome inhibitor MG132. In contrast, inhibitors targeting the autophagy-lysosomal pathway did not have a significant impact on preventing SHBs degradation (Fig. 1A). Given that the ubiquitination-mediated proteasome degradation pathway plays a crucial role in protein degradation, we explored whether SHBs is subject to this modification. By introducing a ubiquitin-expressing plasmid into SHBs-expressing HCC cells, we observed through immunoprecipitation assays that SHBs is indeed modified by ubiquitin (Fig. 1B). Since K48-linked polyubiquitination is known for signaling proteasome-mediated protein degradation, we specifically examined if SHBs undergoes K48-linked polyubiquitination. Our results confirmed that SHBs is polyubiquitinated by K48-linked polyubiquitination (Fig. 1C). Since LHBs and MHBs contain the SHBs domain (20), we investigate whether they are subject to K48-linked polyubiquitination. Our results indicate that both LHBs (Fig. 1D) and MHBs (Fig. 1E) underwent polyubiquitination through K48-linked polyubiquitination chains. Furthermore, HepG2 or Huh7 cells were co-transfected with plasmids encoding 1.2HBV and HA-K48UB, treated with MG132, and subjected to immunoprecipitation using anti-SHBs affinity gel. This analysis revealed that HBsAg, including LHBs, MHBs, and SHBs, was also modified by K48-linked ubiquitin (Fig. 1F). These findings collectively indicate that SHBs is primarily degraded through the proteasome pathway and undergoes K48-linked polyubiquitination.

### K122 is essential for the K48-linked polyubiquitination-dependent degradation of SHBs

**Fig 2:**
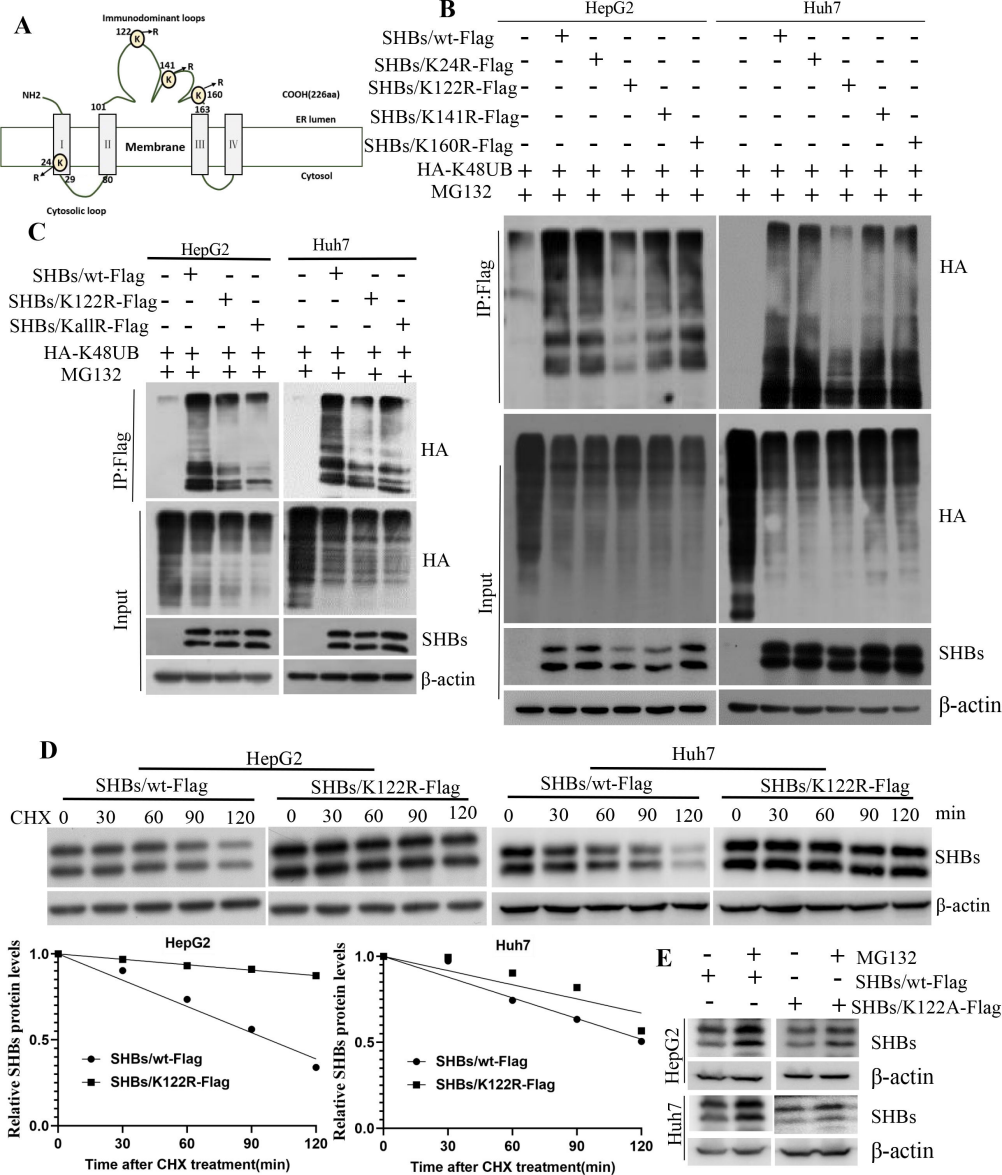
The K122 residue is essential for K48-linked polyubiquitination and subsequent degradation of SHBs. (A) Schematic diagram showing the mutation sites of K residues in SHBs (protein ID: AAD16254.1). (B) In SHBs, K residues at positions 24, 122, 141, and 160 were substituted with R. Following transfection with plasmids encoding either wild-type SHBs or its mutants, cells underwent an 8 hour treatment with MG132 (20 µM) before conducting the ubiquitination assay. (C) Specific mutations were introduced where K122 or all K residues in SHBs were replaced with R as indicated, and subsequent ubiquitination assays were carried out. (D) The stability of wild-type SHBs and its K122R mutant was assessed via Western blot analysis post 100 µg/mL CHX treatment for specified durations, with the accompanying graph depicting the quantification of SHBs protein levels. (E) Specific mutations were introduced by replacing the K122 residues in SHBs with alanine (K122A) as indicated. Cells were transfected with plasmids encoding either wild-type SHBs or K122A and then treated with MG132 (20 µM) for 8 hours before being immunoblotted with an SHBs antibody.

The SHBs protein of the genotype B HBV has four lysine (K) residues (Fig. 2A). To pinpoint the specific site of K48-linked polyubiquitination on SHBs, we created mutants where lysine was replaced with arginine (K to R) at positions K24, K122, K141, and K160. These mutants were introduced into HCC cells along with HA-tagged K48-linked polyubiquitination. Upon assessing SHBs polyubiquitination through immunoblotting, we observed a significant reduction in the polyubiquitination levels of the SHBs/K122R mutant compared to the wild-type SHBs (SHBs/wt), while other mutants showed no notable change in polyubiquitination levels compared to SHBs/wt (Fig. 2B). Further tests were conducted by replacing all K residues in SHBs with R (SHBs/KallR). The polyubiquitination levels of SHBs/KallR were found to be similar to those of the K122R mutant (Fig. 2C), suggesting that K122 is the primary polyubiquitination site of SHBs. To assess the impact of the K122R mutation on SHBs degradation, we performed a cycloheximide (CHX) chase experiment in which Huh7 and HepG2 cells were transfected with plasmids encoding SHBs/wt-Flag or SHBs/K122R-Flag, and then continually exposed to cycloheximide for different time periods up to 120 min. The half-life of the SHBs protein in Huh7 and HepG2 cells increased significantly with the SHBs/K122R mutant, from 124 to 181 min and 98 to 475 min, respectively (Fig. 2D). To further investigate, we substituted the K122 residue in SHBs with alanine (SHBs/K122A) and observed that, in the presence of the proteasomal inhibitor MG132, the SHBs/K122A mutant showed only a slight increase in SHBs levels, whereas the wild-type SHBs exhibited a substantial elevation of the protein level (Fig. 2E). From these findings, we concluded that the K122 site is crucial for the ubiquitin-proteasome-mediated degradation of SHBs.

### Identification of ubiquitin-modifying enzymes interacting with SHBs

Post-translational modification of proteins is caused by the interaction between modifying enzymes and protein substrates (21). We initially introduced a K48UB-expressing plasmid into Huh7 cells that were also expressing SHBs, followed by treatment with the proteasome inhibitor MG132 to block the proteolytic activity of the 26S proteasome complex. The cell lysates underwent immunoprecipitation using FLAG M2 affinity gel, after which the precipitated proteins were subjected to SDS-PAGE. This was followed by in-gel tryptic digestion, and the resulting peptides were analyzed using liquid chromatography-tandem mass spectrometry (LC-MS/MS) (Fig. S1A). This LC-MS/MS analysis revealed a list of 1,726 proteins potentially binding to SHBs. Among these, we focused on searching for E3 ubiquitin ligases and deubiquitinases. Currently, there are more than 650 known E3 ubiquitin ligases and over 100 deubiquitinases (22). By intersecting E3 ubiquitin ligases or deubiquitinases with proteins potentially binding to SHBs, we identified two E3 ubiquitin ligases and five deubiquitinases that may bind to SHBs (Fig. S1B, left panel). TRIM21 was distinguished by 31 unique peptides and achieved a “sum PEP score” of 127.878, while OTUD4 was characterized by 30 unique peptides, accumulating a “sum PEP score” of 177.79 (Fig. S1B, right table). This resulted in identifying TRIM21, an E3 ubiquitin ligase, and OTUD4, a deubiquitinase, as potential interactors with SHBs, as depicted in Fig. S1C’s MS/MS spectra (TRIM21 on the top and OTUD4 on the bottom).

### The interaction between SHBs and the E3 ubiquitin ligase TRIM21

**Fig 3:**
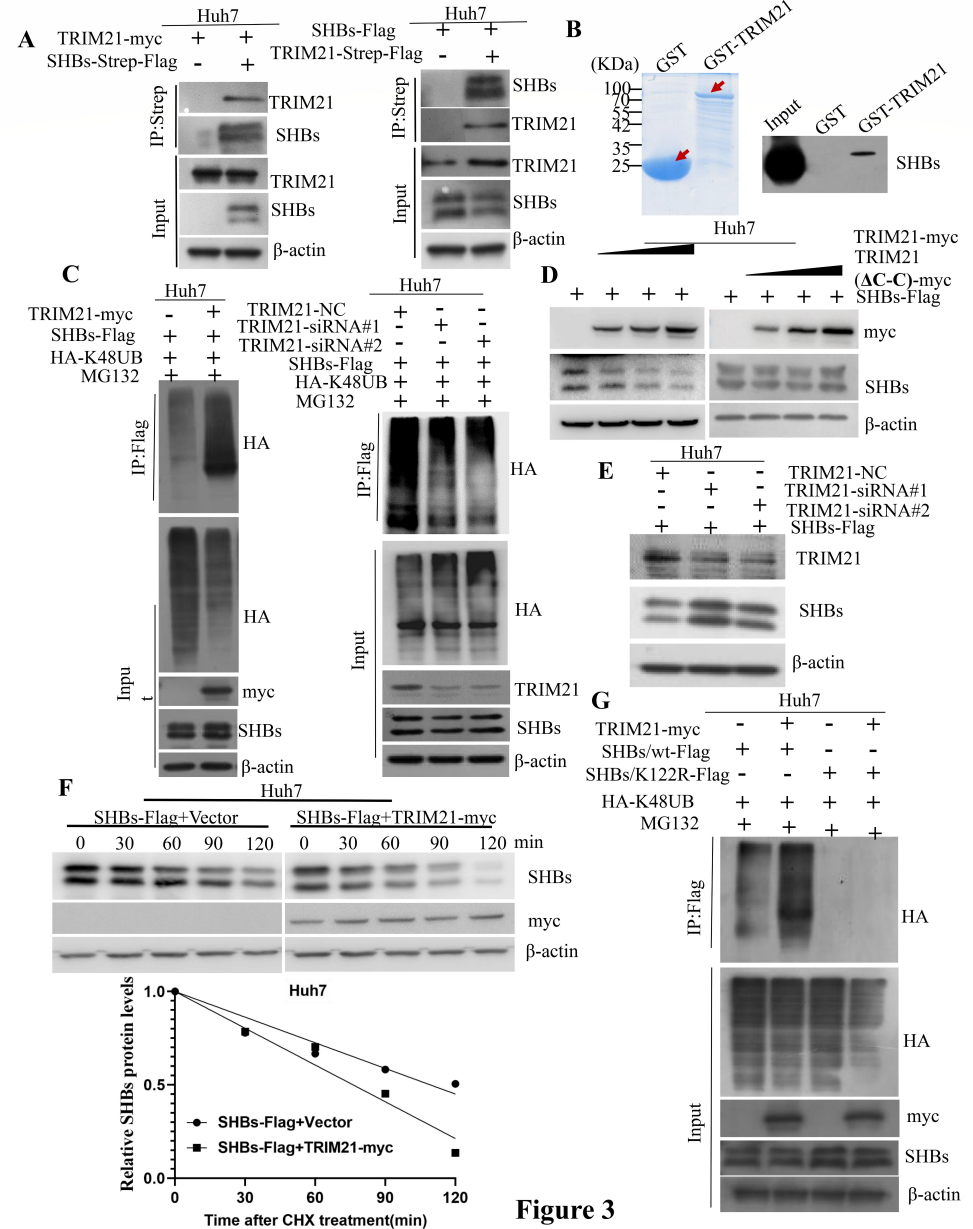
TRIM21 promotes SHBs protein degradation via a ubiquitin/proteasome-dependent pathway. (A) Huh7 cells were co-transfected with plasmids encoding TRIM21-myc and SHBs-Strep-Flag (left panel), or co-transfected with plasmids encoding TRIM21-Strep-Flag and SHBs-Flag (right panel) without MG132 treatment. Forty-eight hours later, the cell lysates were then subjected to immunoprecipitation using Strep-Tactin and analyzed by immunoblotting with antibodies specific to TRIM21 (left) and SHBs (right). (B) *In vitro* translated SHBs protein was mixed with GST-tagged TRIM21, followed by a GST pull-down assay and subsequent immunoblotting using anti-SHBs. (C) Huh7 cells were co-transfected with plasmids encoding SHBs-Flag, HA-K48UB, and TRIM21-myc (left panel) or siRNA knocking down TRIM21 (right panel), followed by an 8-hour MG132 (20 µM) treatment, and the cell lysates were immunoprecipitated with anti-FLAG M2 affinity gel and analyzed via immunoblotting with the appropriate antibodies. (D) Huh7 cells were co-transfected with plasmids encoding SHBs-Flag and increasing amounts of wild-type TRIM21 or its deletion mutant (ΔC-C). The cell lysates were then harvested for immunoblotting with specific antibodies. (E) Immunoblotting with designated antibodies was performed on lysates from Huh7 cells transfected with plasmids encoding SHBs-Flag and siRNA targeting TRIM21. (F) Following co-transfection with plasmids encoding SHBs-Flag and TRIM21-myc (or the control vector pCDNA3.1/myc-His(-)A) for 48 hours and subsequent CHX (200 µg/mL) treatment at set intervals, Huh7 cells were immunoblotted with SHBs and myc antibodies, with a graph depicting SHBs protein level quantification. (G) Ubiquitination assays were conducted on lysates from cells co-transfected with plasmids encoding TRIM21-myc, HA-K48UB, and either wild-type SHBs or its K122R mutant for 40 hours, followed by an 8 hour MG132 (20 µM) treatment.

We introduced a plasmid expressing SHBs-Strep-Flag alongside a TRIM21-myc expressing plasmid into Huh7 cells. Coimmunoprecipitation was conducted utilizing an anti-Strep antibody, while an anti-TRIM21 antibody was used to confirm TRIM21’s presence in the immunoprecipitation complex, verifying the interaction between SHBs and TRIM21 (Fig. 3A, left). Additionally, co-transfection of plasmids expressing TRIM21-Strep-Flag and SHBs-Flag into Huh7 cells, followed by immunoprecipitation using an anti-Strep antibody and detection with an anti-SHBs antibody, further substantiated the interaction between SHBs and TRIM21 (Fig. 3A, right). Moreover, we generated a plasmid to express TRIM21 with a GST tag and found that GST-tagged TRIM21 could successfully precipitate *in vitro* translated SHBs in a GST pull-down assay (Fig. 3B). Since TRIM21 is known to have four domains (RING, B-Box, coiled-coil, and PRY-SPRY, Fig. S2A) (23), we created deletion mutants for each domain to determine which one binds to SHBs. The results from these GST pull-down assays indicated that the coiled-coil domain of TRIM21 is crucial for its interaction with SHBs (Fig. S2B).

### TRIM21 regulates the degradation of SHBs via the ubiquitin-proteasome pathway

As an E3 ubiquitin ligase, TRIM21 plays a role in promoting the ubiquitin-proteasome degradation of proteins (24). To elucidate the regulatory effect of TRIM21 on SHBs, HepG2 or Huh7 cells were co-transfected with plasmids encoding TRIM21-myc and SHBs-Flag along with HA-K48UB, in the presence of MG132. The overexpression of TRIM21 significantly enhanced the polyubiquitination levels of SHBs (Fig. 3C; Fig. S3A, left panel). Additionally, using two specific small interfering RNAs (siRNAs) to silence TRIM21, we observed that SHBs polyubiquitination decreased upon TRIM21 knockdown (Fig. 3C; Fig. S3A, right panel). To further assess TRIM21’s effect on SHBs protein levels, we either overexpressed TRIM21 or knocked it down with siRNA in HCC cells. The results showed that SHBs protein levels progressively declined with increasing amounts of TRIM21 in a dose-dependent manner (Fig. 3D; Fig. S3B, left panel). Conversely, reducing TRIM21 levels significantly elevated SHBs protein levels (Fig. 3E; Fig. S3C). However, TRIM21 mutants lacking the coiled-coil domain did not affect SHBs protein levels (Fig. 3D; Fig. S3B, right panel).

We then evaluated the impact of TRIM21 on SHBs stability in cells treated with cycloheximide at different intervals. Elevating TRIM21 levels led to a marked reduction in SHBs’ half-life, decreasing from 98 to 69 min in Huh7 cells and from 109 to 76 min in HepG2 cells (Fig. 3F; Fig. S3D). Given the role of K122 as a polyubiquitination site on SHBs, we investigated if TRIM21’s mediation of SHBs polyubiquitination occurs through the K122 site by comparing the polyubiquitination of SHBs/K122R mutant with that of SHBs/wt. The results showed that TRIM21 increased the polyubiquitination of SHBs/wt, whereas it had no such effect on the SHBs/K122R mutant (Fig. 3G). These results imply that TRIM21 could facilitate the polyubiquitination and subsequent proteasomal degradation of SHBs via the K122 site, and the coiled-coil domain of TRIM21 is crucial for its influence on SHBs protein levels.

### The interaction between SHBs and the deubiquitinase OTUD4

**Fig 4:**
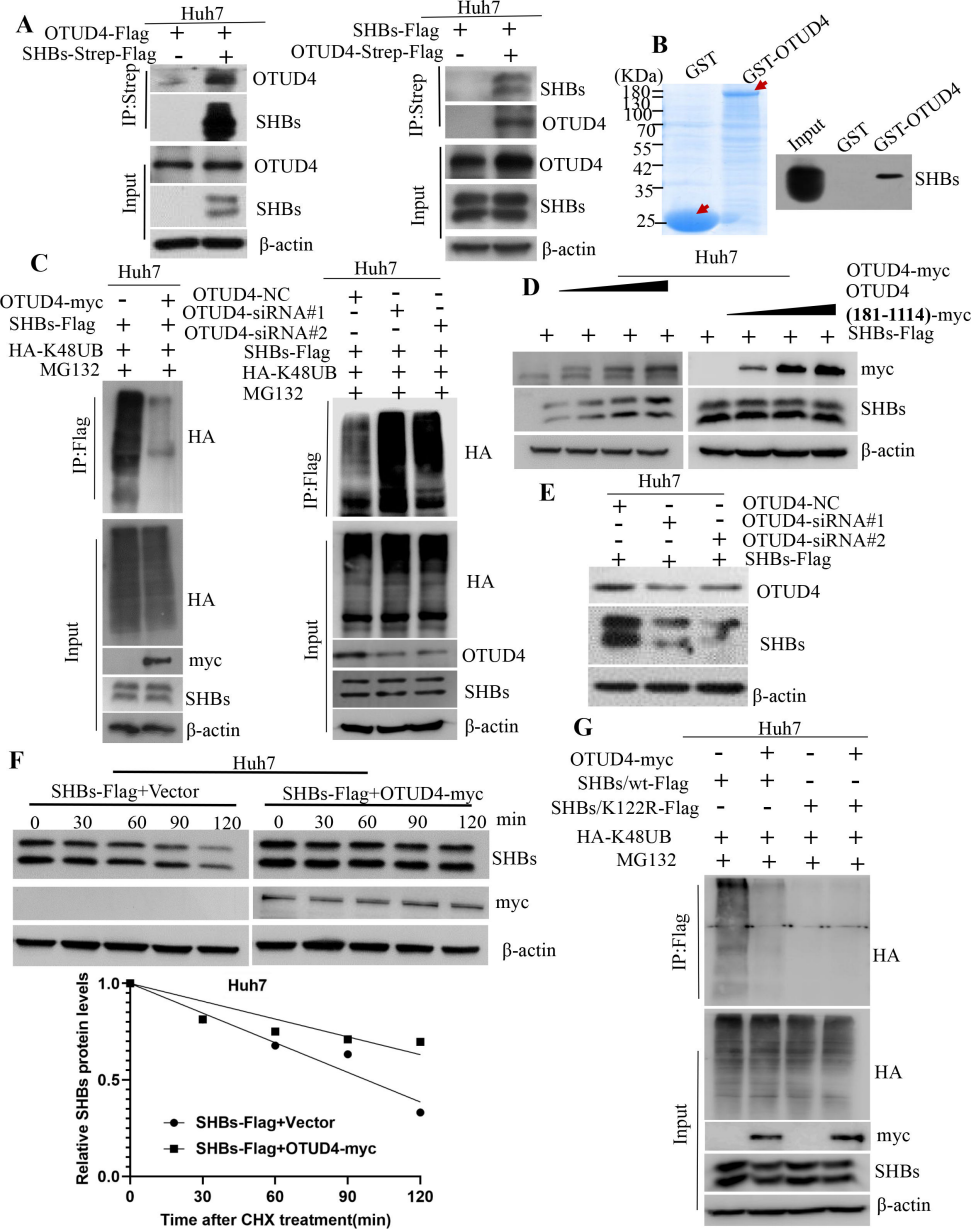
OTUD4 attenuates SHBs protein degradation in a ubiquitin/proteasome-dependent manner. (A) Huh7 cells were co-transfected with plasmids encoding OTUD4-Flag and SHBs-Strep-Flag (left panel), or co-transfected with plasmids encoding OTUD4-Strep-Flag and SHBs-Flag (right panel) in the absence of MG132 treatment. Forty-eight hours later, the cell lysates were then subjected to immunoprecipitation using Strep-Tactin and analyzed by immunoblotting with antibodies specific to OTUD4 (left) and SHBs (right). (B) *In vitro* translated SHBs protein was mixed with GST-tagged OTUD4, then subjected to a GST pull-down assay and analyzed by immunoblotting with an SHBs-specific antibody. (C) Huh7 cells were co-transfected with plasmids encoding SHBs-Flag, HA-K48UB, and OTUD4-myc (left panel) or siRNA knocking down OTUD4 (right panel) followed by an 8 hour MG132 (20 µM) treatment. The cell lysates underwent a ubiquitination assay. (D) Huh7 cells co-transfected with plasmids encoding SHBs-Flag and either wild-type OTUD4 or its fragment (residues 181–1114) in a dose-dependent manner had their lysates immunoblotted with specified antibodies. (E) The lysates from Huh7 cells transfected with plasmid encoding SHBs-Flag and siRNA targeting OTUD4 were immunoblotted with the indicated antibodies. (F) Huh7 cells were co-transfected with plasmids encoding SHBs-Flag and OTUD4-myc (or the pCDNA3.1/myc-His(-)A vector), followed by CHX (200 µg/mL) treatment at specified times. The lysates were analyzed using a half-life assay, with a graph depicting SHBs protein level quantification. (G) Huh7 cells co-transfected with plasmids encoding OTUD4-myc, HA-K48UB, and wild-type SHBs or its K122R mutant, followed by 8 hours of MG132 (20 µM) treatment, had their lysates assessed using a ubiquitination assay.

OTUD4, known for its role in stabilizing proteins and as a K48-specific deubiquitinase (25), was investigated for its potential interaction with SHBs. To verify an interaction between OTUD4 and SHBs, Huh7 cells were co-transfected with plasmids encoding SHBs-Strep-Flag and OTUD4-Flag. Following immunoprecipitation with an anti-Strep antibody, OTUD4 was detected in the resulting complex. Moreover, after co-transfecting with plasmids encoding OTUD4-Strep-Flag and SHBs-Flag into Huh7 cells, immunoprecipitation with the anti-Strep antibody also revealed the presence of SHBs in the precipitated complex. This coimmunoprecipitation assay further confirmed the interaction between OTUD4 and SHBs (Fig. 4A). Moreover, *in vitro* GST pull-down assays revealed that SHBs-Flag directly interacts with GST-OTUD4 (Fig. 4B). To identify which part of OTUD4 is involved in this interaction, we constructed a series of OTUD4 deletion mutants (Fig. S4A) as previously described (26). The interaction of these mutants with SHBs was assessed through GST pull-down assays, indicating that the region comprising amino acids 1–180 of OTUD4 is both necessary and sufficient for binding to SHBs (Fig. S4B).

### OTUD4 regulates the degradation of SHBs via the ubiquitin-proteasome pathway

As a member of the ovarian tumor (OTU) family and an enzyme that cleaves K48-linked polyubiquitin chains, OTUD4’s impact on SHBs was a key focus of our study. We investigated how OTUD4 affects SHBs by examining the polyubiquitination of SHBs under conditions of both overexpression and knockdown of OTUD4, confirmed through immunoprecipitation assays. Our results indicated that increased levels of OTUD4 significantly reduced the polyubiquitination of SHBs (Fig. 4C; Fig. S5A, left panel), whereas silencing OTUD4 led to an increase in SHBs polyubiquitination (Fig. 4C; Fig. S5A, right panel). We also found that boosting OTUD4 levels notably raised the protein levels of SHBs in a dose-dependent manner. This increase was not observed when the residues 1–180 of OTUD4 were absent (Fig. 4D; Fig. S5B). Conversely, reducing OTUD4 with siRNA led to decreased SHBs protein levels (Fig. 4E; Fig. S5C). To assess whether OTUD4 also impacts the stability of SHBs, we co-transfected with plasmids encoding OTUD4-myc and SHBs-Flag into Huh7 cells and treated these cells with cycloheximide at various intervals. We found that the overexpression of OTUD4 significantly prolonged the half-life of SHBs, increasing it from 97 to 162 min in Huh7 cells and from 89 to 204 min in HepG2 cells (Fig. 4F; Fig. S5D). We next examined whether OTUD4 affects SHBs polyubiquitination at the K122 site by comparing the polyubiquitination levels of the SHBs/K122R mutant and SHBs/wt under OTUD4’s influence. The results demonstrated that OTUD4 reduced the polyubiquitination of SHBs/wt but did not impact the SHBs/K122R mutant (Fig. 4G). These results suggest that OTUD4 increases SHBs stability through its deubiquitinating activity, and the residues 1 to 180 of OTUD4 are essential for its effect on SHBs protein levels.

### OTUD4 counteracts SHBs ubiquitination by competing with TRIM21

**Fig 5:**
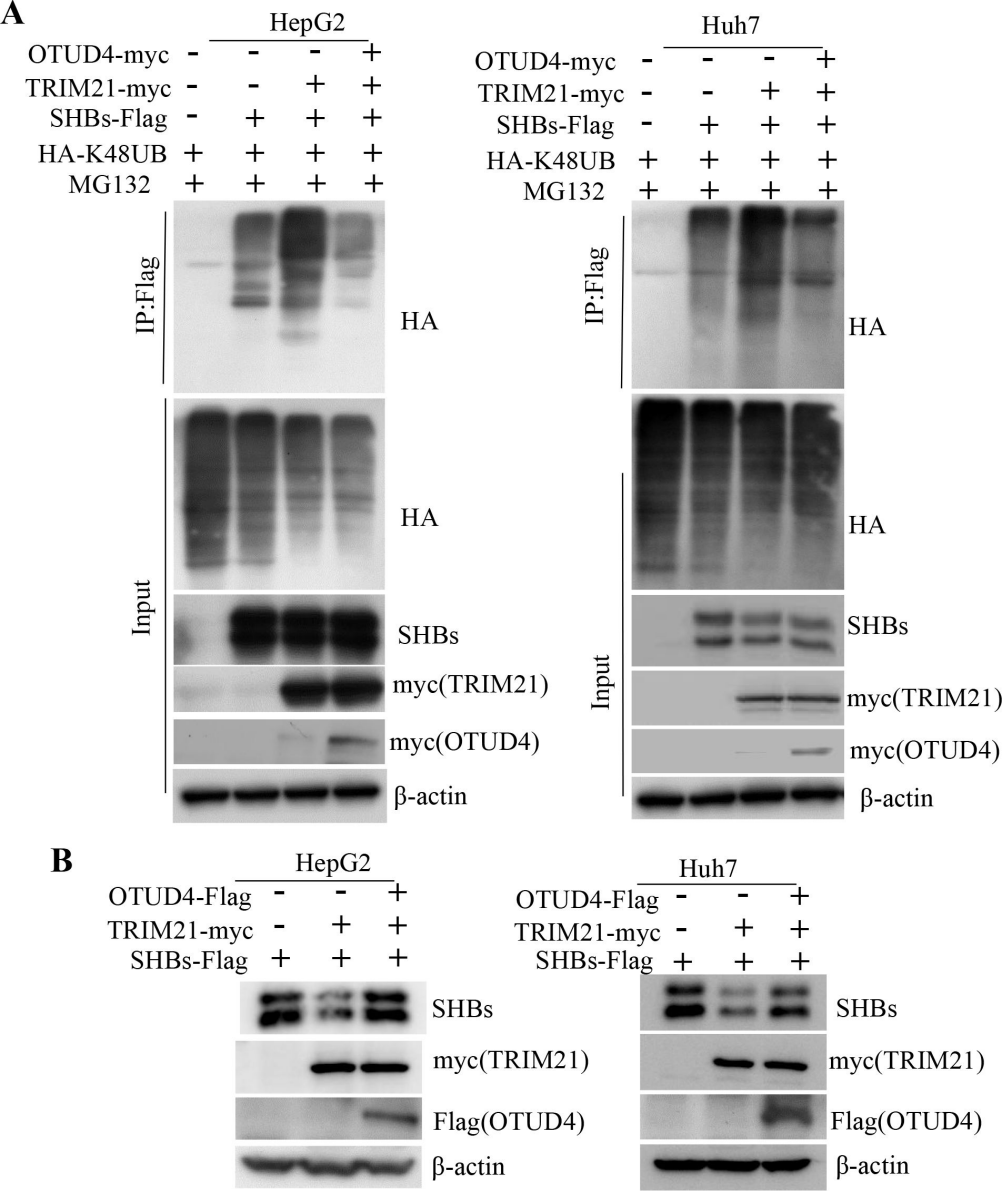
OTUD4 counteracts SHBs ubiquitination by competing with TRIM21. (A) Cells were co-transfected with plasmids encoding HA-K48UB, SHBs-Flag, and TRIM21-myc, either with or without OTUD4-myc, followed by 8 hours of MG132 (20 µM) treatment, after which lysates were analyzed using a ubiquitination assay. (B) Lysates from cells co-transfected with plasmids encoding SHBs-Flag and TRIM21-myc, either with or without OTUD4-Flag, were subjected to immunoblotting using myc, Flag, and SHBs antibodies.

TRIM21, functioning as an E3 ubiquitin ligase, and OTUD4, acting as a deubiquitinase, both play pivotal roles in the degradation process of the SHBs protein. Based on their opposing functions, we theorized that OTUD4 might engage in a competitive interaction with TRIM21, specifically influencing the polyubiquitination and stability of SHBs. This theory posits that OTUD4 could potentially offset the actions of TRIM21, thereby affecting the fate of the SHBs protein within the cell. In line with this hypothesis, our experimental results revealed that when OTUD4 was overexpressed, there was a significant reduction in the polyubiquitination levels of SHBs, which are typically enhanced by TRIM21 (Fig. 5A). This suggests that OTUD4 effectively counteracts the ubiquitination induced by TRIM21. Furthermore, we observed that the overexpression of OTUD4 not only decreased the polyubiquitination but also lessened the reduction in SHBs protein levels that is usually prompted by TRIM21 (Fig. 5B). These results support the notion that OTUD4 acts in opposition to TRIM21, specifically influencing SHBs stability by reducing its polyubiquitination.

### Ubiquitination of SHBs affects its oncogenic activities

**Fig 6:**
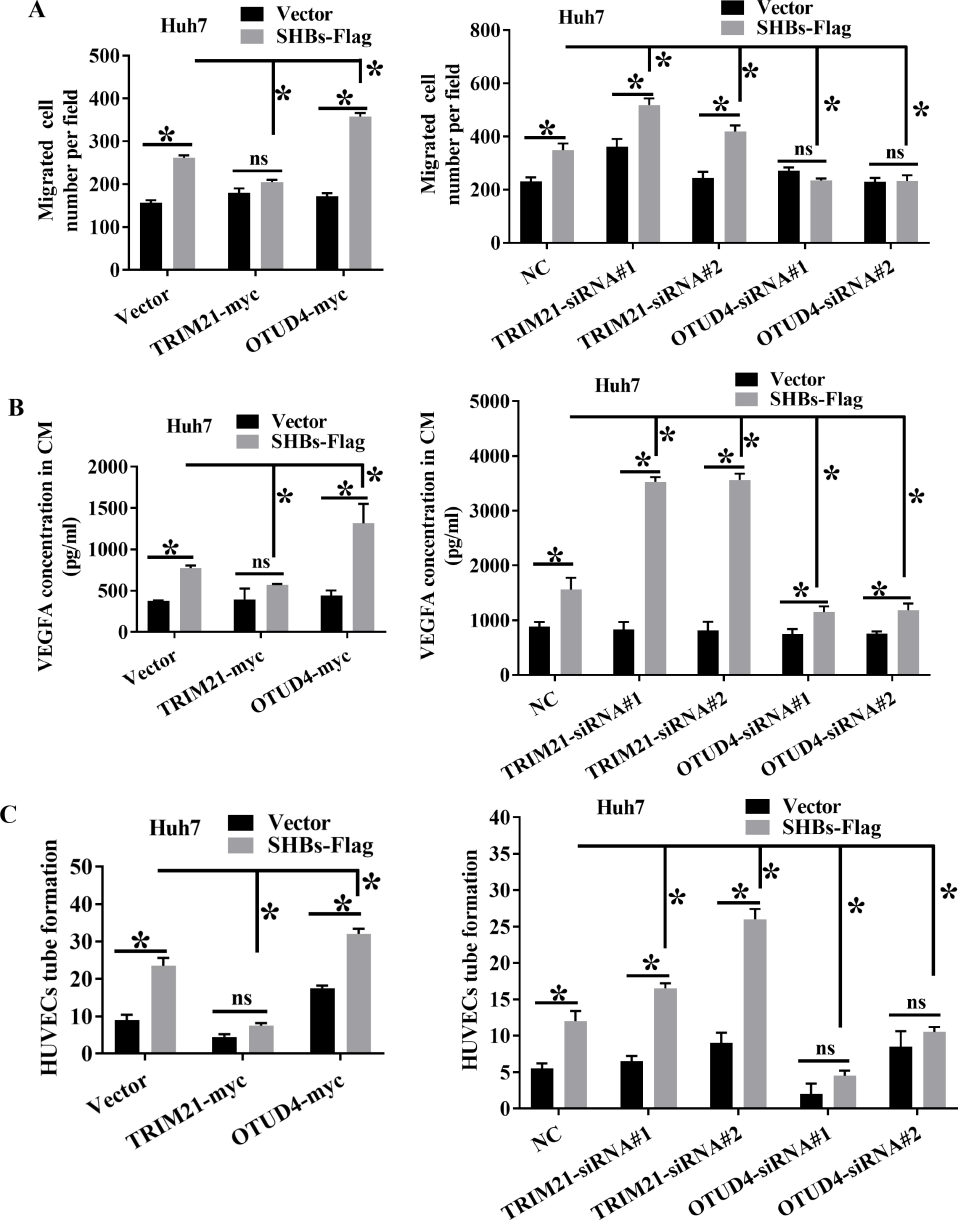
Impact of OTUD4 and TRIM21 on SHBs-associated functions. (A) Transwell migration assays were performed to assess the migration of Huh7 cells co-transfected with plasmids encoding SHBs-Flag (or the control vector pCDNA3.1/myc-His(-)A) and either TRIM21-myc (or OTUD4-myc) or siRNAs targeting TRIM21 (or OTUD4). (B) Enzyme-linked immunosorbent assay was conducted to measure VEGFA protein levels in supernatants from the aforementioned cell cultures. (C) Capillary tube formation assays were carried out with HUVECs cultured in conditioned media (CM) derived from Huh7 cells that were co-transfected with a SHBs-Flag-expressing plasmid (or the control vector pCDNA3.1/myc-His(-)A) and a plasmid expressing TRIM21-myc (or OTUD4-myc) or siRNAs targeting TRIM21 (or OTUD4).

Our prior study has established that SHBs enhances HCC cell migration and angiogenesis by upregulating vascular endothelial growth factor A (VEGFA) expression (10, 11). Given the observation that TRIM21 and OTUD4 can interact with SHBs to affect its stability, we proceeded to investigate whether the biological functions of SHBs could be altered as a result of changes in the SHBs protein level. We transfected Huh7 cells with plasmids encoding SHBs-Flag and OTUD4-myc or TRIM21-myc. As anticipated, introducing TRIM21 substantially decreased the migratory abilities of SHBs-expressing Huh7 cells, as measured by a transwell migration assay. Conversely, OTUD4 overexpression enhanced the capacity of SHBs to promote cell migration (Fig. 6A, left panel). Furthermore, when examining the effects of SHBs on angiogenesis, we found that the culture medium from TRIM21 overexpressed SHBs-expressing Huh7 cells demonstrated a significant decrease in SHBs’ capability to upregulate VEGFA expression and tube formation in human umbilical vein endothelial cells (HUVECs) compared to the control cells. Conversely, overexpressing OTUD4 resulted in an enhanced capability of SHBs to elevate VEGFA expression and promote tube formation (Fig. 6B and C, left panels). Reversing the expression levels of TRIM21 or OTUD4 using siRNA knockdown yielded opposite results (Fig. 6A through C, right panels). These findings suggest that TRIM21 and OTUD4 can alter the functions of SHBs by regulating its protein levels, thereby affecting Huh7 cell migration and the angiogenic ability of HUVECs. This underscores their potential significance in the pathogenesis of SHBs-associated HCC.

### TRIM21 and OTUD4 affect subviral particle and virion production

**Fig 7:**
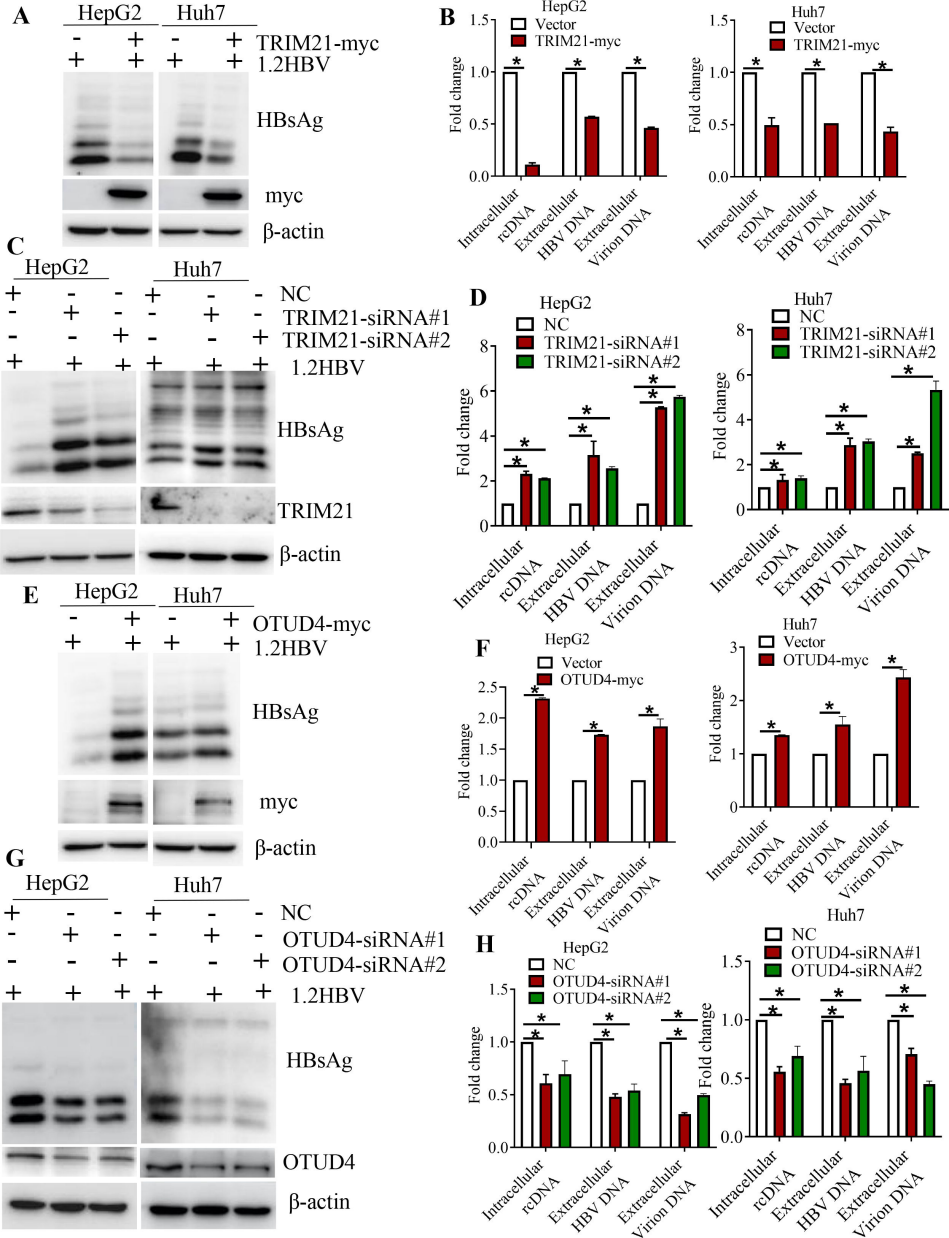
OTUD4 and TRIM21 alter the production of subviral particles and virions. (A, B, E, F) Lysates were prepared from Huh7 and HepG2 cells co-transfected with plasmids encoding 1.2HBV and either TRIM21-myc (**A, B**) or OTUD4-myc (**E, F**). These lysates were subjected to immunoblotting using antibodies against myc, β-actin, and SHBs, and intracellular HBV DNA levels were quantified by qPCR. Additionally, cell culture supernatants were analyzed by qPCR to quantify extracellular HBV DNA and virion DNA levels. (C, D, G, H) Lysates from cells transfected with plasmids encoding 1.2HBV and siRNA targeting either TRIM21 (**C, D**) or OTUD4 (**G, H**) were similarly processed. The lysates were immunoblotted using the indicated antibodies, and intracellular HBV DNA was quantified by qPCR. qPCR was performed on the supernatants to measure HBV DNA and virion DNA levels.

HBsAg is crucial for immune response, HBV infection, virion production, and potential HBV cure (27). To evaluate the impact of TRIM21 and OTUD4 on HBsAg and virion production, a series of experiments were performed. The results demonstrated that overexpressing TRIM21 in Huh7 and HepG2 cells led to a decrease in both intracellular HBsAg and HBV DNA, as well as a marked reduction in extracellular HBsAg, HBeAg, and titer of HBV virions (Fig. 7A and B; Fig. S6A). In contrast, knocking down TRIM21 in HCC cells resulted in significantly elevated levels of intracellular and extracellular HBsAg, HBV DNA, and increased extracellular HBeAg compared to the control group (Fig. 7C and D; Fig. S6C). Additionally, overexpression of OTUD4 caused an increase in intracellular HBsAg, HBV DNA, as well as extracellular HBsAg, HBeAg, and HBV virion DNA (Fig. 7E and F; Fig. S6C). Conversely, silencing OTUD4 expression with siRNA led to a decrease in these markers (Fig. 7G and H; Fig. S6D). Similar findings were obtained in HepG2.215 that express HBV genotype D (Fig. S7A through H). These results suggest that the regulation of HBV production by TRIM21 and OTUD4 may involve effects on both HBV replication and the secretory process. This regulation could be linked to the well-documented fact that increased SHBs expression can induce ER stress, promoting autophagy and enhancing HBV replication (9, 28).

## DISCUSSION

Even with the availability of HBV vaccine, chronic HBV infection remains a significant global health issue, affecting over 296 million people and leading to an estimated 820,000 deaths (1). Achieving HBsAg loss 24 weeks after therapy cessation is now considered a “functional cure” for chronic HBV, yet this outcome is rare with current treatments (29). To address HBsAg clearance, various therapeutic strategies have been developed. Small-interfering RNAs (like AR520) and antisense oligonucleotides are designed to hinder the synthesis or translation of HBsAg. However, a rapid reduction in HBsAg levels can lead to induced elevations in alanine aminotransferase (29). Furthermore, while RNA interference (RNAi) and antibody-mediated HBsAg clearance can reduce HBV viral load and alleviate immune dysfunction, they do not effectively activate virus-specific T cells for long-term viral control. As a result, only a small percentage of patients develop anti-HBsAg antibodies, and viral antigens often reappear after discontinuation of the treatment (30). Additionally, nucleic acid polymers (NAPs) and S antigen traffic-inhibiting oligonucleotide polymers (STOPs) have been utilized to reduce HBsAg secretion, a key approach in current HBsAg clearance strategies (5). Although NAPs and STOPs rapidly reduce circulating HBsAg, they have minimal effect on other viral markers. Therefore, investigating novel methods for HBsAg clearance remains crucial in the fight against chronic HBV.

Ubiquitination is a crucial process in the replication and pathogenesis of HBV. For instance, the cullin-RING ligase 4 targets the HBV polymerase protein (Pol) for ubiquitination and proteasome degradation, effectively hindering HBV replication (17). Similarly, the HBc undergoes ubiquitination by the NIRF, leading to its proteasomal degradation and reduced virus particle release (18). Additionally, the HBx is ubiquitinated and degraded by Siah-1, diminishing its transcriptional activity (31). SHBs, as the most abundant viral protein, surpasses the levels of MHBs and LHBs, with subviral particles mainly composed of SHBs being 1,000 to 10,000 times more abundant than the actual HBV Dane particles (32). Clearing SHBs could thus be a key strategy in reducing HBsAg levels in patients with chronic HBV. However, the specific degradation pathway of SHBs protein has been largely unknown. In our study, we demonstrate that SHBs is predominantly degraded via the proteasome pathway (Fig. 1), aligning with recent findings (33). Although it was previously reported that SHBs protein undergoes ubiquitination-independent degradation by 20S proteasomes (33), our research reveals that SHBs is subject to K48-linked polyubiquitination at the K122 residue, resulting in proteasome-dependent degradation. Similarly, both LHBs and MHBs are also subject to K48-linked polyubiquitination (Fig. 2). We further confirmed that the ubiquitination and stability of SHBs are influenced by E3 ubiquitin ligases and deubiquitination processes (Fig. 3 and 4). The reason for the discrepancies observed is not entirely clear. One possible explanation might be the different substitutions of K residues in the SHBs protein. In the study by Guo et al. (33), the SHBs protein had its K residues substituted with alanine, and all four lysine residues of the SHBs protein were replaced with alanine. However, in our investigation, these lysine residues were replaced with arginine (R). Additionally, we also tested the phenotype of the K122A mutant by replacing the K122 residue in SHBs with alanine (SHBs/K122A) and found that in the presence of the proteasomal inhibitor MG132, only a slight elevation in SHBs levels was observed for the K122A mutant compared to a marked increase in SHBs levels for the wild-type SHBs. Our findings strongly suggest that the ubiquitin-dependent proteasome pathway plays a crucial role in regulating SHBs stability. Deciphering the mechanisms and biological implications of SHBs ubiquitination might contribute to strategies for SHBs clearance, potentially offering a functional cure for chronic HBV infection.

E3 ubiquitin ligases and deubiquitination processes are essential for the ubiquitination of proteins. In this study, we utilized immunoprecipitation combined with LC-MS/MS to identify proteins that interact with SHBs and influence its ubiquitination. Our findings revealed that SHBs directly binds to the E3 ubiquitin ligase TRIM21 and is deubiquitinated by OTUD4. TRIM21, a protein in the tripartite motif-containing (TRIM) family and an E3 ubiquitin ligase, comprises several key motifs and domains: a RING motif essential for E3 ligase activity, a B-box motif and a coiled-coil domain crucial for TRIM21 oligomerization and interaction with other proteins to form macromolecular complexes, and a PRY-SPRY domain that allows binding to various proteins, contributing to functional diversity (23). It is involved in various diseases, including cancer development and response to cancer drugs, making it a potential target for cancer therapy (34, 35). Additionally, TRIM21 plays several roles in viral infections. It can influence immune and inflammatory responses and degrade viral proteins (36). For example, TRIM21 targets a protein called IRF3 for degradation through its SPRY domain, leading to a decrease in the production of type I interferon, which is dependent on IRF3 (37). Furthermore, TRIM21-mediated polyubiquitination and proteasomal degradation of SAMHD1 affects its ability to combat viruses (24). In the context of the virus, it interacts with the porcine epidemic diarrhea virus and degrades its nucleocapsid protein, which hinders virus proliferation (38), and binds to the TP domain of Pol or HBx through its SPRY domain, leading to the ubiquitination and degradation of these viral proteins, thereby suppressing HBV replication (16, 39). While previous studies reported that TRIM21 strongly interacts with HBx and has weak binding to the HBc protein, but not with HBsAg (39), our research demonstrated that TRIM21 binds to SHBs through its coiled-coil domain (Fig. S2). This interaction enhances the ubiquitination of SHBs at the K122 site, leading to its degradation by the proteasome (Fig. 3). The discrepancies between these findings and previous reports may be due to different HBV genotypes. In the study by Xu et al. (39), the SHBs protein encoded by the HBV genotype D has an R at position 122, while in our study, the SHBs protein encoded by the HBV genotype B has a K at this position. Most of the SHBs proteins encoded by HBV genotypes A, B, or C have lysine at position 122, whereas genotype D has arginine (data not shown). Our findings using the 1.2-mer construct of genotype B and the HepG2.2.15 cell line (stably transfected with a genotype D clone) indicate that the regulatory effects of TRIM21 and OTUD4 on HBV are conserved across different HBV genotypes. This suggests that the functional differences between K122 (genotype B) and R122 (genotype D) do not play a genotype-specific role in the context of full-length HBV replication and protein stability. Further investigation into the impact of TRIM21 and OTUD4 overexpression or silencing in various HBV genotypes is warranted. Such studies could provide critical insights into their potential as broad-spectrum antiviral targets for HBV therapeutic development. We observed that both LHBs and MHBs are also subject to K48-linked polyubiquitination (Fig. 2). However, Liu et al. demonstrated that the degradation of MHBs occurs in a ubiquitination-independent manner (40). One possible explanation for this discrepancy could be that the MHBs protein in Liu et al.’s study was encoded by HBV genotype D, whereas in our study, the MHBs protein is encoded by HBV genotype B. Furthermore, Liu et al. introduced an R122K mutation in the genotype D S protein and found that while MHBs undergoes K122-dependent ubiquitination, this modification does not affect its stability. In contrast, our study demonstrated that MHBs in genotype B undergoes K48-linked polyubiquitination, although its precise impact on protein stability requires further investigation. Our findings shed light on the molecular mechanism underlying the regulation of SHBs stability and offer insights into how TRIM21-mediated ubiquitination serves as a critical modulator in the proteasomal degradation pathway, potentially opening new avenues for targeted therapeutic strategies in related disorders.

Deubiquitinating enzymes are capable of reversing the ubiquitination process. OTUD4, a K48-specific deubiquitinase, features an OTU catalytic domain essential for its deubiquitinating activity and a deubiquitinase recruiting domain (26). Viral infections have been noted to induce OTUD4 expression via IRF3/7, which subsequently binds to mitochondrial antiviral signaling protein (MAVS), inhibiting its ubiquitination. This inhibition stabilizes MAVS, thereby bolstering the body’s innate antiviral defenses (25). Furthermore, OTUD4 interacts with the Kaposi’s sarcoma-associated herpesvirus (KSHV) replication and transcription activator (K-RTA), increasing K-RTA stability through deubiquitination, which in turn facilitates KSHV reactivation (41). Yet, the role of OTUD4 in the deubiquitination of viral proteins, particularly SHBs, has been less clear. Our research reveals that SHBs interacts with the OTU catalytic domain (residues 1–180) of OTUD4 (Fig. S4). This interaction inhibits K48-linked polyubiquitination of SHBs, leading to reduced degradation via the proteasome pathway (Fig. 4). This study is among the first to illustrate that OTUD4 plays a role in regulating the ubiquitination of a viral protein, thereby influencing HBV pathogenesis and virion productivity (Fig. 6 and 7). It has been reported that deubiquitinases can compete with E3 ubiquitin ligases to bind substrates, thereby exerting a deubiquitinating effect. Ubiquitin-specific protease 17 can compete with the E3 ubiquitin ligase murine double minute 2 to bind the target protein p53, maintaining the stability of p53 and its downstream signaling pathways (42). The cylindromatosis protein, which contains the OTU domain, can act as a deubiquitinase to compete with the E3 ubiquitin ligase tripartite motif protein 25 (TRIM25) for binding to the retinoic acid-induced gene protein I (RIG-I), thereby negatively regulating RIG-I-mediated signal transduction (43). Our findings highlight the dynamic interplay between TRIM21, which acts as an E3 ligase, and OTUD4, functioning as a deubiquitinase, in the process of SHBs ubiquitination. We discovered that OTUD4 competes with TRIM21, stabilizing SHBs through deubiquitination (Fig. 5). However, the specific mechanisms by which OTUD4 counteracts TRIM21’s role in promoting ubiquitination and reducing SHBs stability require further investigation. This interplay represents a complex and nuanced aspect of viral protein regulation that could have significant implications for understanding and treating viral infections.

SHBs, which is pivotal in the HBV life cycle and HBV-related liver diseases (44), has been shown in our previous study to promote angiogenesis and metastatic progression (10, 11). In our current study, we demonstrated that increasing the expression of TRIM21 and decreasing the activity of OTUD4 effectively suppress angiogenesis and invasiveness in HCC cells driven by SHBs. This approach also reduces the levels of HBsAg and other viral markers, such as HBeAg and viral titer. Conversely, reducing TRIM21 levels and enhancing OTUD4 activity lead to an increased ability of SHBs to promote angiogenic and aggressive processes, along with higher expression of HBsAg and other viral markers, including HBeAg and viral titer. Notably, the effects of TRIM21 and OTUD4 on HBeAg levels cannot be solely attributed to their regulation of HBsAg polyubiquitination. Instead, these changes may be mediated through the degradation of HBx, a key transcriptional activator of HBV mRNAs. Additionally, alterations in intracellular replicative HBV DNA levels are unlikely to be a direct consequence of changes in intracellular envelope protein expression but rather linked to the degradation of P protein (16, 39). Our findings indicate that TRIM21 and OTUD4 regulate SHBs, HBx, and Pol proteins via the ubiquitin-dependent proteasomal degradation pathway. Therefore, targeting this pathway with OTUD4 inhibitors and TRIM21 activators may represent a promising antiviral strategy for HBV-related diseases.

Overall, our results highlight the roles of TRIM21 as an E3 ligase and OTUD4 as a deubiquitinase in regulating the stability of SHBs within the ubiquitin-dependent proteasome pathway. This understanding might provide a solid foundation for developing strategies aimed at SHBs elimination and achieving a functional cure for HBV in clinical practice.

## MATERIALS AND METHODS

### Cell lines and culture

The human hepatoma cell lines HepG2 and Huh7 were sourced from the American Type Culture Collection (Manassas, VA, USA) and the Japanese Collection of Research Bioresources Cell Bank (Osaka, Japan), respectively, while HUVECs were generously provided by Dr. Huang (45). The HepG2.215 cells (abbreviated as 2.2.15 hereafter) were cultured as previously described (46). The cell lines Huh7 and HepG2 were cultured in Dulbecco’s Modified Eagle Medium (DMEM; Invitrogen, Carlsbad, CA, USA) and Minimum Essential Medium (Invitrogen), respectively. HUVECs were maintained in endothelial cell medium (ScienCell, Carlsbad, CA, USA). All cultures included 10% heat-inactivated fetal bovine serum (FBS; HyClone, Logan, UT, USA) and were incubated in a 5% CO_2_ humidified atmosphere at 37°C.

### Plasmid construction

The pRep-1.2HBV and its empty control, pREP10, along with the plasmids pCDNA3.1/myc-His(-)A, pCDNA3.1-Strep-Flag, pCDNA3.1-SHBs-Flag (validated in Fig. S8 to retain SHBs functionality despite the fusion tag), pCDNA3.1-HA-UB, and pCDNA3.1-HA-K48UB, have mutations at all lysine residues except for K48 of UB, as described previously (10, 19). To construct pCDNA3.1-SHBs-Strep-Flag, the SHBs gene, which contains only the S domain, was PCR-amplified from HBV DNA (3215 bp, genotype B, adw subtype, GenBank accession number: AF100309) and inserted into pCDNA3.1-Strep-Flag. The LHBs gene, containing pre-S1, pre-S2, and S domains (with the MHBs and SHBs start codons, ATG, mutated to ACG), and the MHBs gene, containing pre-S2 and S domains (with the SHBs start codon ATG mutated to ACG), were chemically synthesized by General Biosystems and inserted into pCDNA3.1/myc-His(-)A to construct pCDNA3.1-LHBs-Flag and pCDNA3.1-MHBs-Flag, respectively. Mutant constructs pCDNA3.1-SHBs/K24R-Flag, pCDNA3.1-SHBs/K122R-Flag, pCDNA3.1-SHBs/K141R-Flag, pCDNA3.1-SHBs/K160R-Flag, pCDNA3.1-SHBs/KallR-Flag, and pCDNA3.1-SHBs/K122A-Flag, expressing various SHBs mutations, were generated by inserting chemically synthesized SHBs mutants into pCDNA3.1/myc-His(-)A. OTUD4 (GenBank accession number: NM_001366057.1) and TRIM21 (GenBank accession number: NM_003141.4) genes, tagged with GST, were synthesized by General Biosystems and inserted into pGEX-4T-1 (GE Healthcare, #28-9545-49). pCDNA3.1-TRIM21-Strep-Flag and pCDNA3.1-OTUD4-Strep-Flag, as well as pCDNA3.1-TRIM21-myc and pCDNA3.1-OTUD4-myc, were created by cloning PCR-amplified TRIM21 or OTUD4 genes into pCDNA3.1-Strep-Flag or pCDNA3.1/myc-His(-)A, utilizing pGEX-4T-1-TRIM21 or pGEX-4T-1-OTUD4 as templates. pCMVTNT-SHBs-Flag was assembled by inserting PCR-amplified SHBs gene into pCMVTNT Vector (Promega, #L5620) using XhoI and KpnI sites. Additionally, various TRIM21 or OTUD4 deletion mutants were cloned into pGEX-4T-1. Transfections were conducted with Lipofectamine 3000 (Invitrogen) following the manufacturer’s protocols.

### RNA interference

siRNAs aimed at silencing TRIM21 and OTUD4, along with a control non-targeting siRNA negative control (NC), were custom-designed and synthesized by Shanghai GenePharma Co., China. The siRNAs were introduced into cells using Lipofectamine 3000 (Invitrogen), following the manufacturer’s guidelines. The success of the knockdown was verified through Western blot analysis 48 hours after transfection. siRNA sequences were as follows: NC: 5′-UUCUCCGAACGUGUCACGUTT-3′; TRIM21 siRNA#1: 5′-CCUGUUCUGUGAGA AAGAU TT-3′; TRIM21 siRNA#2: 5′-GCAGAGCAUACCUGGAAAUTT-3′; OTUD4 siRNA#1: 5′-UCGAGAGAACAGAGAGAAATT-3′; OTUD4 siRNA#2: 5′-GGGUAGGACAAGUGGA AAU TT-3′.

### Western blot analysis

For Western blot analysis, cells were lysed in Western and immunoprecipitation (IP) lysis buffer containing 20 mM Tris (pH 7.5), 150 mM NaCl, 1% Triton X-100, sodium pyrophosphate, β-glycerophosphate, EDTA, Na3VO4, and leupeptin (Beyotime Biotechnology, Shanghai, China) with added protease inhibitors including a cocktail and phenylmethylsulfonyl fluoride (PMSF) (MedChemExpress, USA) for 30 min at 4°C. The BCA Protein Assay Kit (Vazyme Biotech Co., China) was used to measure protein concentrations as per the manufacturer’s instructions. Proteins were then separated on a 12% SDS-PAGE gel under reducing conditions and transferred onto polyvinylidene fluoride (PVDF) membranes (Bio-Rad Laboratories, USA). Membranes were blocked with Tris-buffered saline (TBS)-Tween 20 containing 5% bovine serum albumin (BSA) and incubated overnight at 4°C with primary antibodies against HBsAg (Creative-Diagnostics, #DMABT-51328MH), OTUD4 (abcam, #ab106368), β-actin (#4970S), HA (#3724S), TRIM21 (#92043S), and myc (#2276S) sourced from Cell Signaling Technology. Following thorough washes, horseradish peroxidase (HRP)-coupled secondary antibody (Cell Signaling Technology) was applied for an hour. Protein bands were visualized using BeyoECL Star (Beyotime Biotechnology).

### LC-MS/MS

Huh7 cells were transfected with plasmids encoding Flag-tagged SHBs and HA-K48UB for 40 hours, followed by treatment with 20 µM MG132 (MCE, #HY-13259) for 8 hours. Post-treatment, cells were lysed using Western and IP lysis buffer, and the lysates were subjected to overnight immunoprecipitation at 4°C with anti-FLAG M2 affinity gel. The immunoprecipitated complexes were then separated via SDS-PAGE and analyzed using mass spectrometry on an Easy-nLC 1200 system coupled to an Orbitrap Exploris 480 mass spectrometer (Thermo Fisher Scientific). Mass spectrometry data were processed using Proteome Discoverer (version 3.0), and database searches were conducted with the Homo sapiens Uniprot database (https://www.uniprot.org/proteomes/ UP000005640) to identify the proteins.

### Coimmunoprecipitation (Co-IP) assay

In the Co-IP experiments conducted *in vivo*, cellular lysates were prepared, and the soluble protein fraction was incubated overnight with Strep-Tactin XT Superflow high capacity beads (IBA-Lifesciences, #2-5030-002) at 4°C. Following this incubation, the complexes that had been immunoprecipitated were then analyzed through Western blotting to detect and examine the proteins involved.

### GST pull-down assay

In the GST pull-down assay, GST and GST-tagged proteins of TRIM21, OTUD4, and their respective mutants were produced in the *Escherichia coli* strain Rosetta (DE3) and subsequently purified using previously described methods (19). Flag-tagged SHBs proteins were synthesized *in vitro* using the pCMVTNT-SHBs-Flag vector with the T7 Quick Coupled Transcription/Translation System (Promega, #L1171) and semipermeabilized cells following the manufacturer’s instructions. Following synthesis, the SHBs proteins were combined with GST-tagged TRIM21, OTUD4, or their mutant variants, which were pre-bound to glutathione Sepharose 4B beads (GE Healthcare, #17-0756-01). This step facilitates the interaction between the SHBs proteins and the GST-tagged proteins. After the incubation, allowing for protein interactions, the protein complexes that formed on the beads were isolated and then subjected to Western blot analysis. This analysis helps in identifying and characterizing the interactions between SHBs proteins and the GST-tagged TRIM21 or OTUD4 proteins, along with their mutant forms.

### SHBs protein stability assay

Huh7 cells were co-transfected with Flag-tagged SHBs along with either TRIM21-myc or OTUD4-myc for a duration of 40 hours. Subsequently, the cells were exposed to 200 µg/mL CHX (MCE, #HY-12320), a protein synthesis inhibitor, to halt new protein production. The incubation with CHX was conducted over time intervals of 0, 30, 60, 90, and 120 min to monitor the degradation process of the SHBs protein. At each specified time point, cells were harvested and lysed to extract proteins, which were then analyzed using Western blotting. This method allowed for the measurement of SHBs protein levels at different times post-CHX treatment, providing insights into the protein’s half-life and how its stability is influenced by the presence of TRIM21 or OTUD4.

### Ubiquitination assay

Huh7 and HepG2 cells underwent transfection with Flag-tagged SHBs for a duration of 40 hours. Following the transfection period, the cells were treated with 20 µM MG132, a proteasome inhibitor, for 8 hours to prevent the degradation of ubiquitinated proteins. Post-treatment, the cells were lysed using Western and IP lysis buffer, which was enriched with a mix of protease inhibitors including a cocktail and PMSF, for 30 min to ensure efficient protein extraction. The cell lysates were then diluted fivefold with a Western and IP lysis buffer to maintain protein integrity for subsequent steps. These diluted lysates were then subjected to immunoprecipitation using anti-FLAG M2 affinity gel, specifically targeting the Flag-tagged SHBs proteins for isolation. Once the immunoprecipitation process was completed, the isolated proteins were analyzed via western blotting. This analysis aimed to detect the ubiquitination of SHBs proteins, with the presence of ubiquitin conjugates indicating successful ubiquitination.

### *In vitro* migration assays

For the migration assays, cells were placed in the upper chamber of a transwell apparatus (BD Biosciences, USA) at a density of 5 × 10^4^ cells per well, utilizing serum-free media to prevent non-specific cell movement. The lower chamber was filled with DMEM supplemented with 10% FBS to serve as a chemoattractant, encouraging cell migration through the membrane. Following a 24 hour incubation period, cells remaining on the upper side of the membrane, which had not migrated, were carefully wiped away using a cotton swab. This step ensures that only cells that have actively migrated through the membrane are analyzed. Subsequently, the migrated cells on the lower side of the membrane were fixed to preserve their structure and then stained using Giemsa solution. This staining enhances the visibility of the cells for counting and analysis. The counting and examination of the migrated cells were performed using a Qimaging Micropublisher 5.0 RTV microscope camera (Olympus Corporation, Japan), providing a clear and quantifiable measure of cell migration ability under the various experimental conditions.

### VEGFA quantification

The cell culture supernatants were collected, and VEGFA was quantified by the Quantikine VEGFA enzyme-linked immunosorbent assay (ELISA) kit (Abcam, #ab119566), a specialized assay designed for the accurate detection and quantification of VEGFA.

### Tube formation assays

The tube formation assay was conducted using HUVECs, seeded at a density of 1 × 10^4^ cells per well in a 96-well plate. The HUVECs were incubated at 37°C for 6 hours in the conditioned medium obtained from Huh7 cells. This conditioned medium contains factors secreted by Huh7 cells that can influence the angiogenic behavior of the endothelial cells. For quantitative analysis, the number of tubes was counted and quantified across five low-power fields at 100× magnification.

### HBV DNA quantification and ELISA detection

The quantification of HBV DNA in the culture supernatant of HBV-transfected cells was performed using the hepatitis B viral DNA quantitative fluorescence diagnostic kit (Santure Biotech, China), following the manufacturer’s instructions. The expression levels of HBsAg or HBeAg in the culture supernatant were measured using human HBsAg or HBeAg ELISA kits (Andy Gene, China), according to the protocols provided by the manufacturer.

### Extraction of virion DNA and qPCR

The quantification of virion DNA in the culture supernatant of HBV-transfected cells was performed using previously described methods (47). HBV virions were immunoprecipitated by preclearing the re-dissolved polyethylene glycol (PEG) precipitates and then capturing the virions with anti-HBsAg antibodies pre-absorbed onto protein A/G-sepharose beads (Santa Cruz, USA). To remove residual transfected plasmid vector DNA, the samples were treated with DNase I. Virion DNAs were then released by proteinase K digestion and precipitated with ethanol in the presence of 10 µg glycogen (TaKaRa) as a carrier. Viral DNA was quantified by qPCR using a hot-start Taq polymerase kit (TaKaRa, catalog no. RR390A) according to the manufacturer’s instructions. The primers used were HBV-fwd 5′-AATGCCCCTATCCTATCAACACT-3′ and HBV-rev 5′-GAGATTGAGA TCTTCTGCGACG-3′, along with a TaqMan probe labeled with FAM at the 5’ end and TAMRA at the 3’ end: 5′-FAM-CCCCTAGAAGAAGAACTCCCTCGCCT-TAMRA-3′. The cycling conditions included an initial denaturation at 95°C for 30 seconds, followed by 40 cycles of denaturation at 95°C for 5 seconds and combined annealing/elongation at 60°C for 25 seconds. The fluorescent signal emitted from the FAM probe, released by Taq polymerase, was measured at the end of each cycle.

### Extraction of intracellular HBV and qPCR

Intracellular HBV DNA was quantified using quantitative PCR, as previously described (48). Briefly, hepatoma cells were lysed using NP-40 lysis buffer (10 mM Tris-HCl [pH 7.9], 50 mM NaCl, 1 mM EDTA, 0.25% Nonidet P-40, protease inhibitor), and the lysates were centrifuged at 14,000 *g* for 5 min to separate the supernatants. To precipitate the viral DNA, 125 µL of 35% PEG 8000 in 1.75 M NaCl was added to the supernatant, and the mixture was incubated on ice for 16 hours. After centrifugation at 12,000 rpm for 10 min, the pellets were re-suspended and treated with DNase I for 30 min at 37°C to ensure the complete digestion of residual transfection DNA. The re-suspended core particles were then digested with proteinase K, and nucleic acids were extracted and precipitated. Finally, qPCR for cytoplasmic viral DNA was performed using a hot-start Taq polymerase kit, following the manufacturer’s protocol.

### Statistical analysis

The statistical evaluation of the data was conducted using SPSS software (SPSS 22.0; Chicago, IL, USA). To analyze protein level variations among different groups, an analysis of variance test was employed. A *P*-value of less than 0.05 was set as the threshold for statistical significance. Each experiment was conducted multiple times independently to ensure the reproducibility and reliability of the results.

## Data Availability

The data sets generated and/or analyzed during the current study are available from the corresponding author on reasonable request.

## SUPPLEMENTAL MATERIAL

Fig. S1 jvi.02309-24-s0001.tif Identification of ubiquitin-modifying enzymes that interact with SHBs.

Fig. S2 jvi.02309-24-s0002.tif Interaction of SHBs with the coiled-coil domain of TRIM21.

Fig. S3 jvi.02309-24-s0003.tif TRIM21 regulates the degradation of SHBs via the ubiquitin-proteasome pathway.

Fig. S4 jvi.02309-24-s0004.tif Interaction of SHBs with residues 1 to 180 of OTUD4.

Fig. S5 jvi.02309-24-s0005.tif OTUD4 regulates the degradation of SHBs via the ubiquitin-proteasome pathway.

Fig. S6 jvi.02309-24-s0006.tif OTUD4 and TRIM21 modulate the production of extracellular HBsAg and HBeAg levels.

Fig. S7 jvi.02309-24-s0007.tif OTUD4 and TRIM21 alter the production of subviral particles and virions.

Fig. S8 jvi.02309-24-s0008.tif Quantification of extracellular SHB levels.

Supplemental legends jvi.02309-24-s0009.docx Legends for Fig. S1 to S8.

ASM does not own the copyrights to Supplemental Material that may be linked to, or accessed through, an article. The authors have granted ASM a non-exclusive, world-wide license to publish the Supplemental Material files. Please contact the corresponding author directly for reuse.
