# Supplementary figures and images for: Ubiquitin-dependent proteasomal degradation of small hepatitis B virus surface antigen mediated by TRIM21 and antagonized by OTUD4
Source: J Virol. 2025 Apr 25;99(5):e02309-24. doi: 10.1128/jvi.02309-24 (PMC12090720; doi:10.1128/jvi.02309-24)

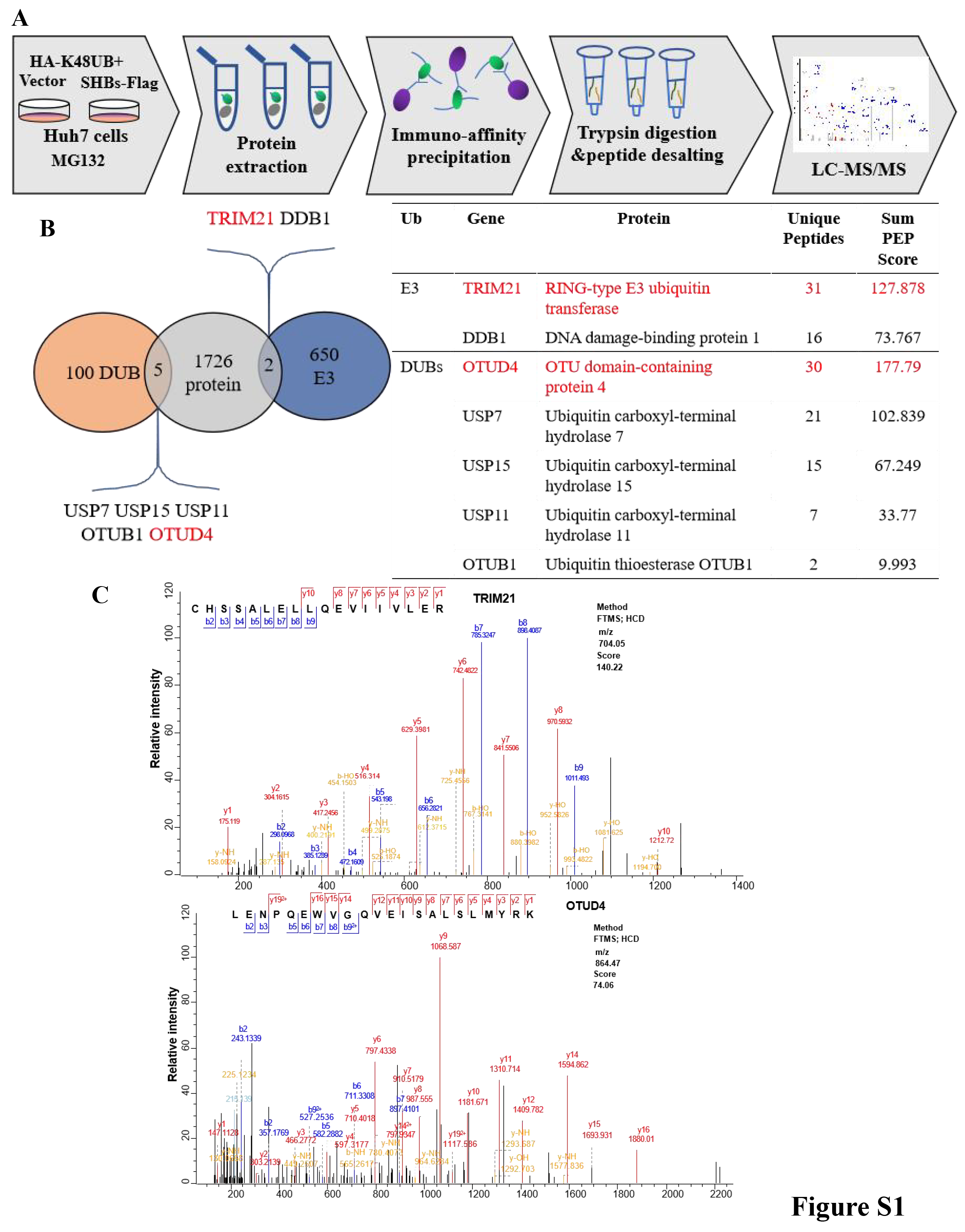

Supplement: Fig. S1 — Identification of ubiquitin-modifying enzymes that interact with SHBs. [file jvi.02309-24-s0001.tif]

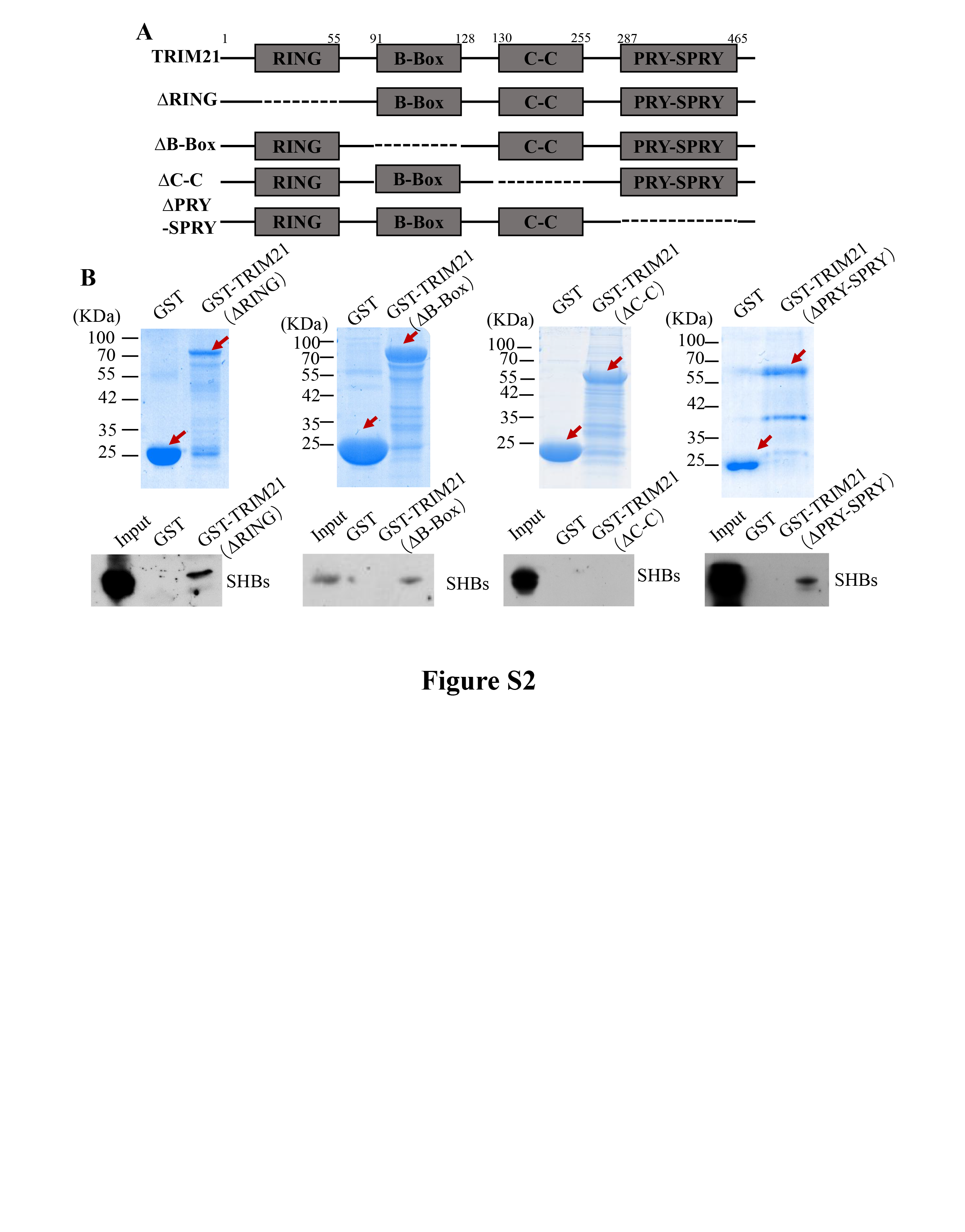

Supplement: Fig. S2 — Interaction of SHBs with the coiled-coil domain of TRIM21. [file jvi.02309-24-s0002.tif]

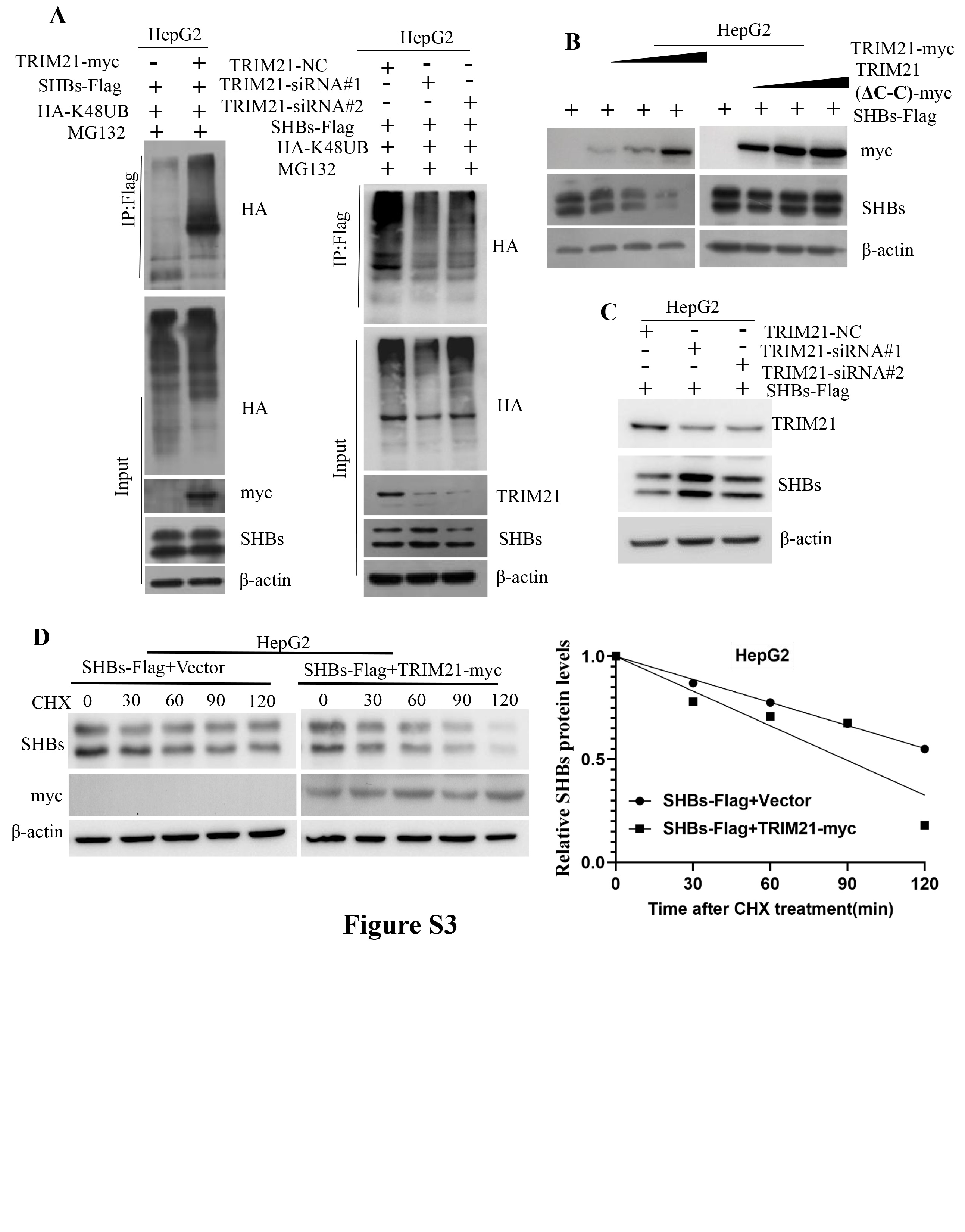

Supplement: Fig. S3 — TRIM21 regulates the degradation of SHBs via the ubiquitin-proteasome pathway. [file jvi.02309-24-s0003.tif]

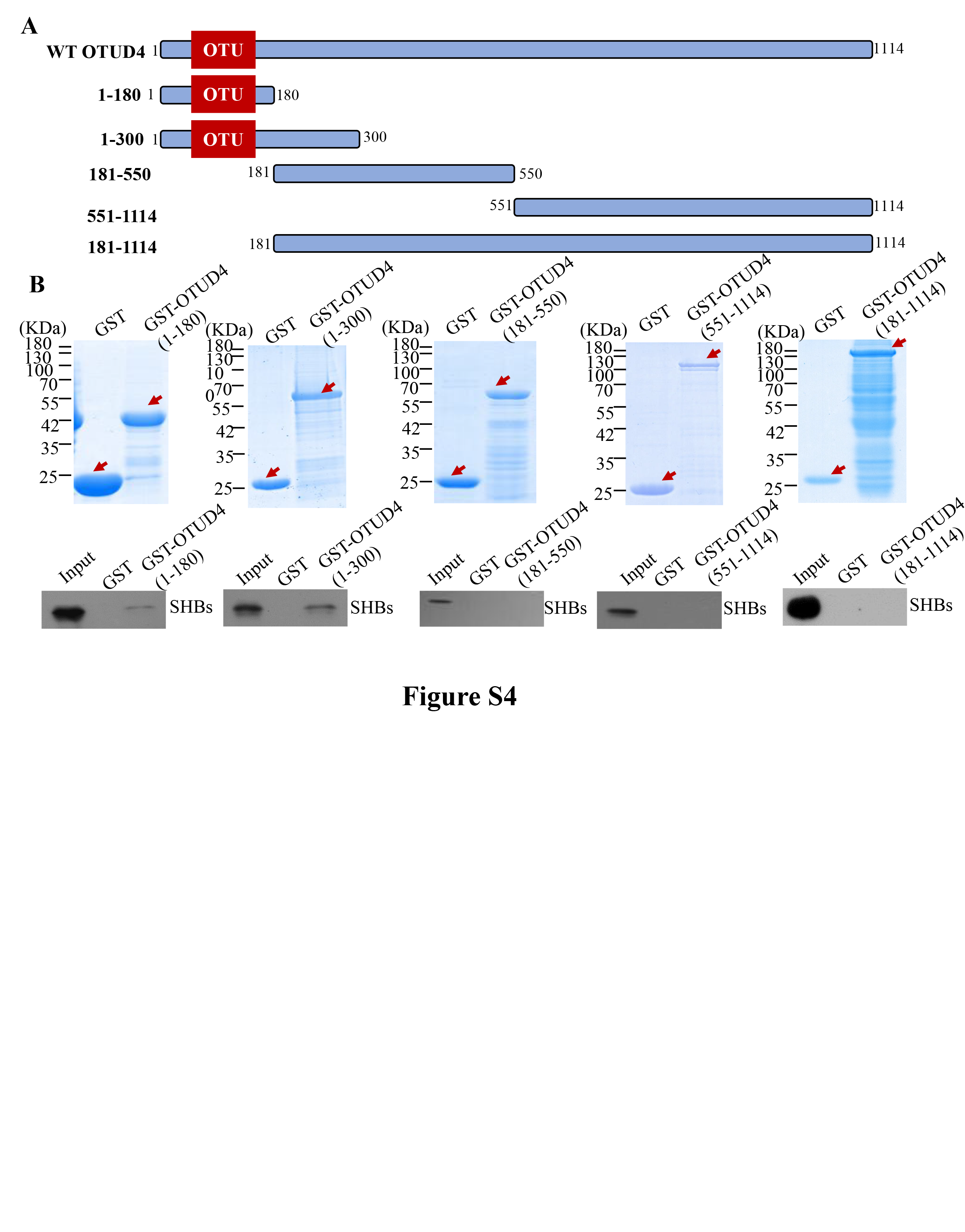

Supplement: Fig. S4 — Interaction of SHBs with residues 1 to 180 of OTUD4. [file jvi.02309-24-s0004.tif]

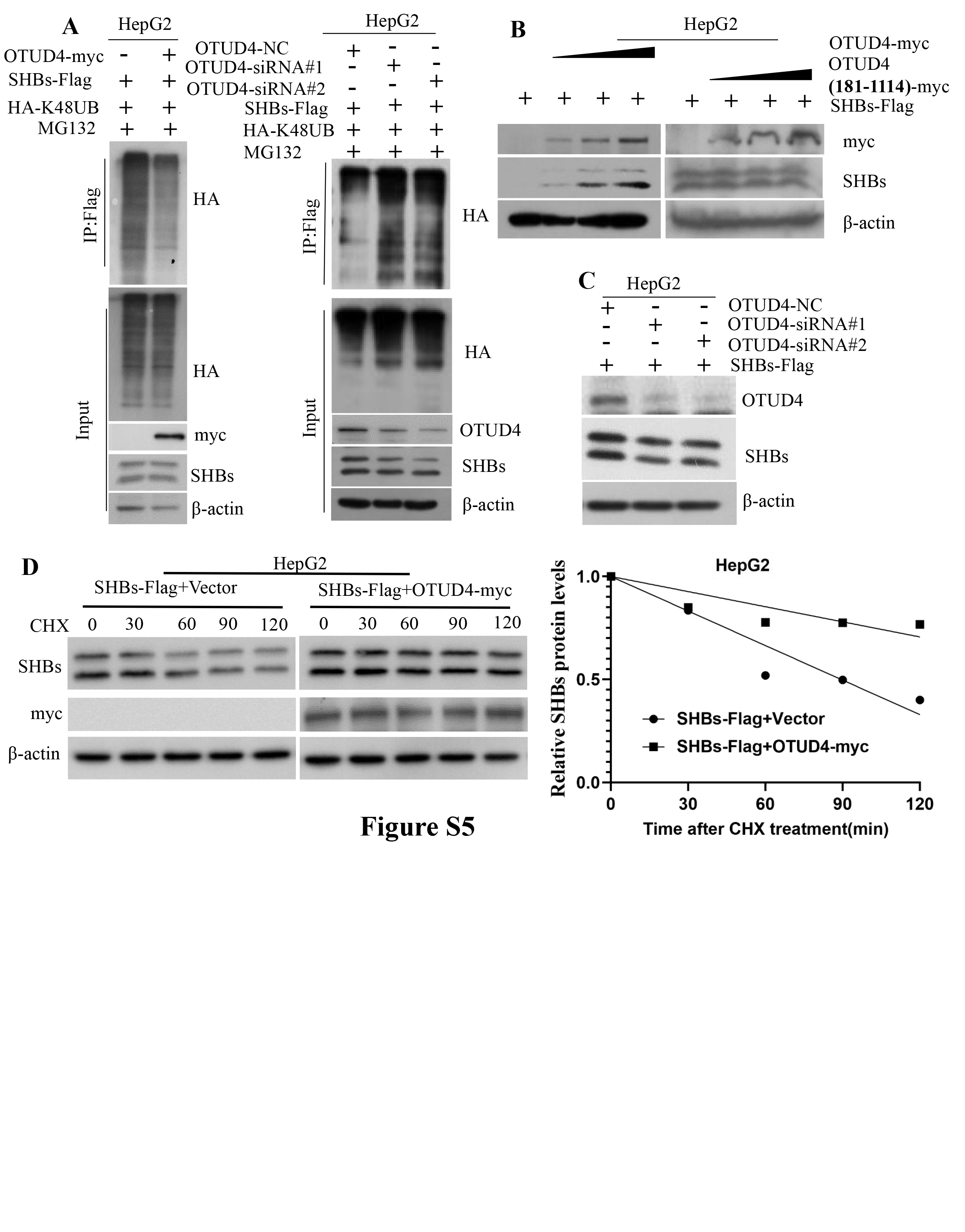

Supplement: Fig. S5 — OTUD4 regulates the degradation of SHBs via the ubiquitin-proteasome pathway. [file jvi.02309-24-s0005.tif]

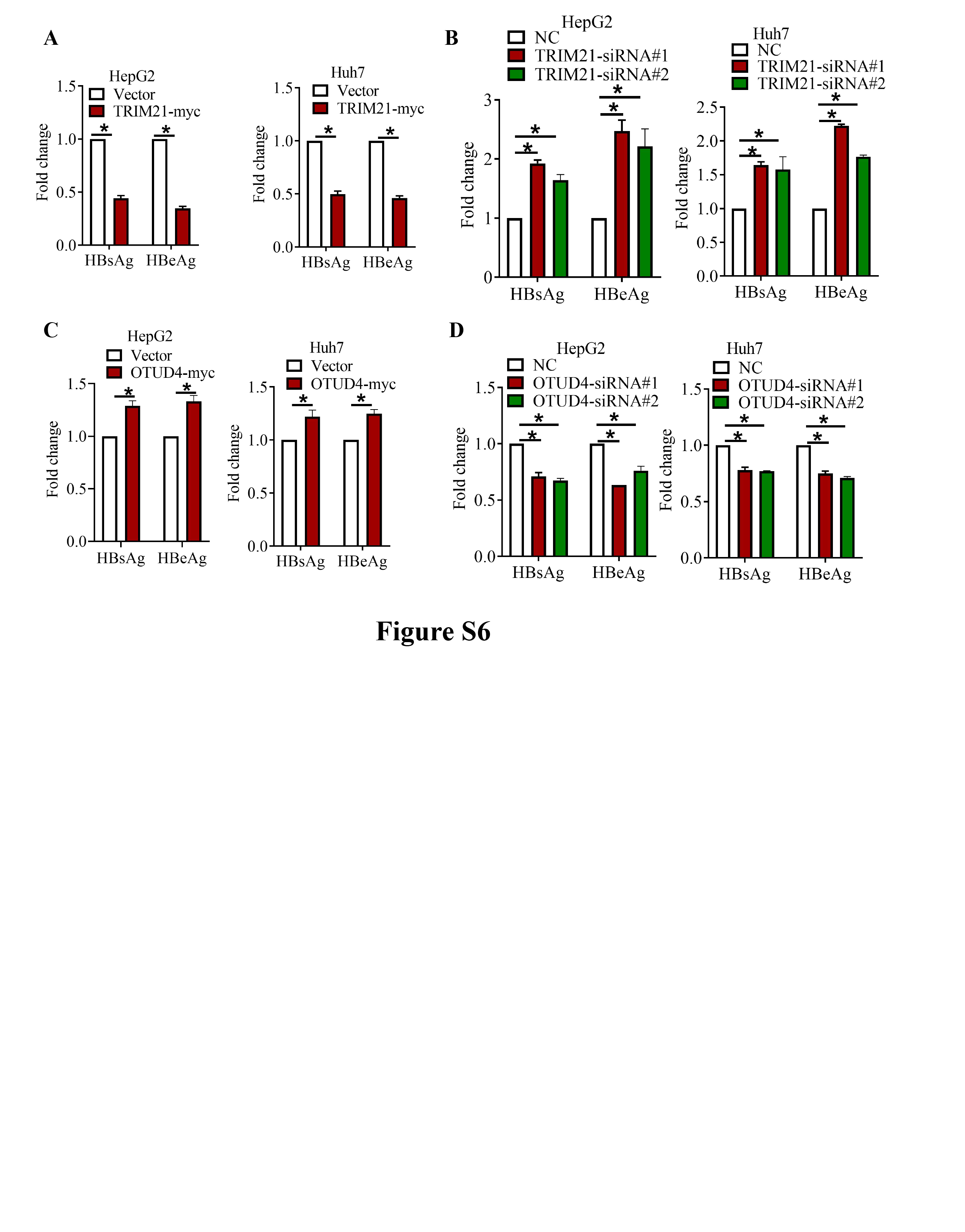

Supplement: Fig. S6 — OTUD4 and TRIM21 modulate the production of extracellular HBsAg and HBeAg levels. [file jvi.02309-24-s0006.tif]

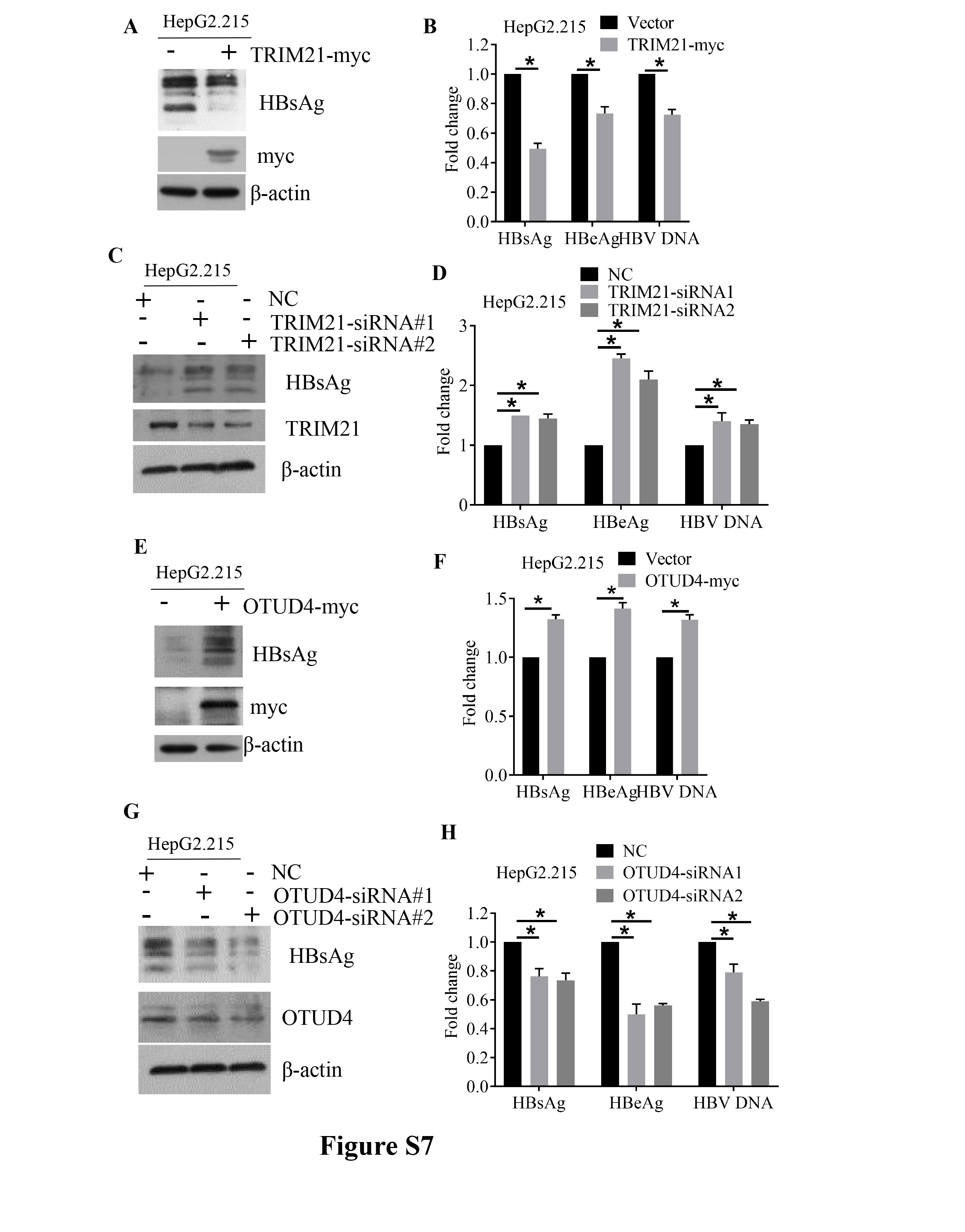

Supplement: Fig. S7 — OTUD4 and TRIM21 alter the production of subviral particles and virions. [file jvi.02309-24-s0007.tif]

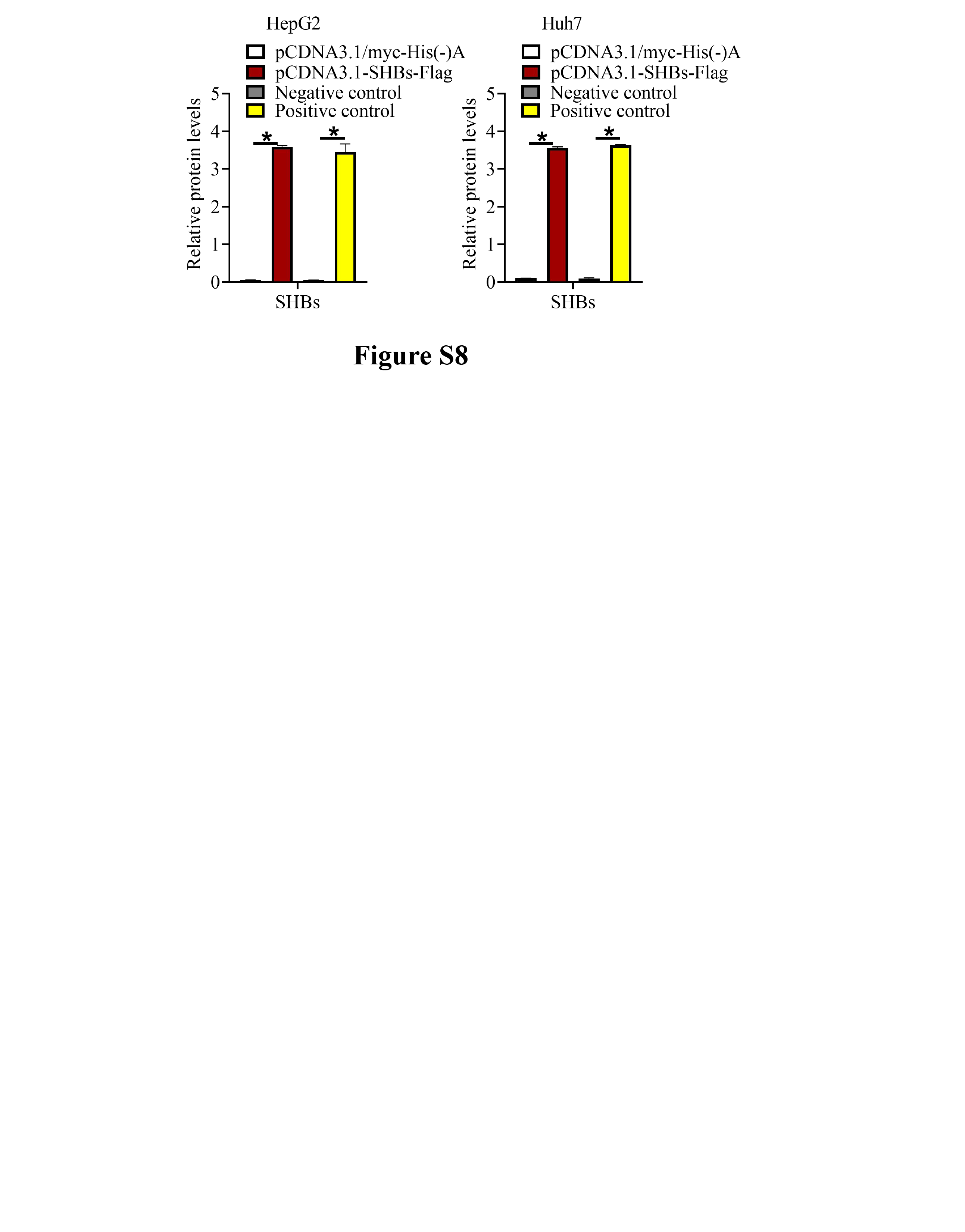

Supplement: Fig. S8 — Quantification of extracellular SHB levels. [file jvi.02309-24-s0008.tif]
