# Supplementary material for: Ubiquitin-dependent proteasomal degradation of small hepatitis B virus surface antigen mediated by TRIM21 and antagonized by OTUD4
Source: J Virol. 2025 Apr 25;99(5):e02309-24. doi: 10.1128/jvi.02309-24 (PMC12090720; doi:10.1128/jvi.02309-24)
Supplement: Supplemental legends — Legends for Fig. S1 to S8. [file jvi.02309-24-s0009.docx]

**SUPPLEMENTARY FIGURE LEGENDS**

**Figure S1. Identification of ubiquitin-modifying enzymes that interact with SHBs. A.** The methodology employed to pinpoint SHBs-binding proteins. **B.** The overlap of potential proteins that bind to SHBs was investigated using liquid chromatography-tandem mass spectrometry (LC-MS/MS), focusing on E3 ubiquitin ligases and deubiquitinases. **C.** Displayed are the MS/MS spectra for TRIM21 (top) and OTUD4 (below).

**Figure S2. Interaction of SHBs with the Coiled-coil domain of TRIM21. A.** schematic illustration of TRIM21 and its various mutants. **B.** Analysis of the interaction between SHBs and various TRIM21 truncation mutants utilizing GST pull-down assays.

**Figure S3. TRIM21 regulates the degradation of SHBs via ubiquitin-proteasome pathway. A.** HepG2 cells were co-transfected with plasmids encoding SHBs-Flag and HA-K48UB, TRIM21-myc (left panel) or siRNA knocking down TRIM21 (right panel), followed by an 8-hour treatment with MG132 (20 µM), the cell lysates were then subjected to ubiquitination assays. B**.** HepG2 cells were co-transfected with plasmids encoding SHBs-Flag and increasing amounts of wild-type TRIM21 or its deletion mutant (ΔC-C), the cell lysates were then harvested for immunoblotting with specific antibodies. **C.** Immunoblotting with designated antibodies was performed on lysates from cells transfected with plasmids encoding SHBs-Flag and siRNA targeting TRIM21. **D.** Following co-transfection with plasmids encoding SHBs-Flag and TRIM21-myc (or the control vector pCDNA3.1/myc-His(-)A) for 48 hours and subsequent CHX (200 µg/ml) treatment at set intervals, HepG2 cells were immunoblotted with SHBs and myc antibodies, with a graph depicting SHBs protein level quantification.

**Figure S4. Interaction of SHBs with residues 1 to 180 of OTUD4.** **A.** A schematic representation of OTUD4 and its various deletion mutants. **B.** Analysis of the interactions between SHBs and different OTUD4 fragments (residues 1-180, 1-300, 181-550, 551-1114, and 181-1114) using GST pull-down assays.

**Figure S5. OTUD4 regulates the degradation of SHBs via ubiquitin-proteasome pathway. A.** HepG2 cells were co-transfected with plasmids encoding SHBs-Flag, HA-K48UB, and OTUD4-myc (left panel) or siRNA knocking down OTUD4 (right panel) followed by an 8-hour MG132 (20 µM) treatment, the cell lysates underwent a ubiquitination assay. **B.** HepG2 cells co-transfected with plasmids encoding SHBs-Flag and either wild-type OTUD4 or its fragment (residues 181-1114) in a dose-dependent manner had their lysates immunoblotted with specified antibodies. **C.** The lysates from HepG2 cells transfected with plasmid encoding SHBs-Flag and siRNA targeting OTUD4 were immunoblotted with the indicated antibodies. **D.** HepG2 cells were co-transfected with plasmids encoding SHBs-Flag and OTUD4-myc (or the pCDNA3.1/myc-His(-)A vector), followed by CHX (200 µg/ml) treatment at specified times, the lysates were analyzed using a half-life assay, with a graph depicting SHBs protein level quantification.

**Figure S6. OTUD4 and TRIM21 modulate the production of extracellular HBsAg and HBeAg levels. A, C.** Cell culture supernatants from Huh7 and HepG2 cells co-transfected with plasmids encoding 1.2HBV and either TRIM21-myc (A) or OTUD4-myc (C) were analyzed by ELISA to quantify extracellular HBsAg and HBeAg levels. **B, D.** ELISA was performed on supernatants from cells transfected with plasmids encoding 1.2HBV and siRNA targeting either TRIM21 (B) or OTUD4 (D) to measure extracellular HBsAg and HBeAg levels.

**Figure S7. OTUD4 and TRIM21 alter the production of subviral particles and virions. A, B, E, F.** Lysates were prepared from HepG2.215 cells transfected with plasmids encoding either TRIM21-myc (A, B) or OTUD4-myc (E, F). Immunoblotting was performed using antibodies against myc, β-actin, and SHBs. Additionally, supernatants were analyzed by qPCR or ELISA to quantify extracellular HBV DNA, HBsAg, and HBeAg levels. **C, D, G, H.** Lysates from cells transfected with siRNA targeting either TRIM21 (C, D) or OTUD4 (G, H) were similarly processed. Immunoblotting was conducted using the indicated antibodies, and qPCR or ELISA were performed on the supernatants to measure HBV DNA, HBsAg, and HBeAg levels.

**Figure S8. Quantification of extracellular SHBs levels.** Huh7 and HepG2 cells were transfected with either the SHBs-Flag expression plasmid or an empty vector control. The culture supernatants were collected and analyzed by ELISA to measure extracellular SHBs levels.
